# Multi-cohort proteogenomic analyses reveal genetic effects across the proteome and diseasome

**DOI:** 10.1016/j.cell.2026.03.049

**Published:** 2026-05-06

**Authors:** Mine Koprulu, Karl Smith-Byrne, Brian Richard Ferolito, Erin Macdonald-Dunlop, Jian’an Luan, Åsa K. Hedman, Chibuzor Franklin Ogamba, Jurgis Kuliesius, Linda Repetto, Anna Ramisch, Fahim Abbasi, Johan Ärnlöv, Themistocles L. Assimes, BeLOVE Study Group, Hanna M. Björck, Sophia Björkander, Morten Böttcher, Adam Stuart Butterworth, Zhengming Chen, Kelly Cho, Robert Joseph Clarke, Simon Riddington Cox, Kamila Czene, John Danesh, George Dedoussis, Sölve Elmståhl, Niclas Eriksson, Per Eriksson, Tõnu Esko, Estonian Biobank Research Team, Aida Ferreiro-Iglesias, Paul William Franks, Jingyuan Fu, J. Michael Gaziano, Mohsen Ghanbari, Christian Gieger, Arthur Gilly, Harald Grallert, Marc James Gunter, Stefan Gustafsson, Andreas Göteson, Per Frans Leonard Hall, Oskar Hansson, Sarah Elizabeth Harris, Caroline Hayward, Christian Herder, Natalia Hernandez-Pacheco, Ziad Hijazi, Robert F. Hillary, Jemma Caroline Hopewell, Shixian Hu, Shih-Jen Hwang, Christina Jern, Åsa Johansson, Lina Jonsson, Anette Kalnapenkis, Nicola Dorothy Kerrison, Pik Fang Kho, Lucija Klaric, Leonhard Kohleick, Julia Kraft, Mikael Landén, Daniel Levy, Liming Li, Lars Lind, Jirong Long, Niklas Mattsson-Carlgren, Erik Melén, Simon Kebede Merid, Philipp Mertins, Karl Michaëlsson, Peter Loof Møller, Federico Murgia, Mette Nyegaard, Young-Chan Park, Ewan Pearson, James Peters, John Ross Petrie, Grace Png, Ozren Polašek, Bram Peter Prins, Stephan Ripke, Michael Roden, Palle Duun Rohde, Saredo Said, SCALLOP Consortium, Xia Shen, Jochen M. Schwenk, Agneta Siegbahn, J. Gustav Smith, Tara M. Stanne, Karsten Suhre, Johan Sundström, Barbara Thorand, Elsa Valdes-Marquez, Costanza L. Vallerga, Joyce B.J. van Meurs, Ana Viñuela, Urmo Võsa, Lars Wallentin, Robin G. Walters, Nicholas John Wareham, Joachim Eduard Weber, Rinse Karel Weersma, James F. Wilson, Simon Winther, Summaira Yasmeen, Daniela Zanetti, Eleftheria Zeggini, Jing Hua Zhao, Alexandra Zhernakova, Daria V. Zhernakova, Matthias Ziehm, Benedikt Mathias Kessler, Alexandre C. Pereira, Anders Mälarstig, Maik Pietzner, Claudia Langenberg

**Affiliations:** 1 Precision Healthcare University Research Institute, Queen Mary University of London, London E1 1HH, UK; 2 MRC Epidemiology Unit, School of Clinical Medicine, University of Cambridge, Cambridge CB2 0QQ, UK; 3 Computational Medicine, Berlin Institute of Health at Charité - Universitätsmedizin Berlin, Berlin 10117, Germany; 4 Cancer Epidemiology Unit, Nuffield Department of Population Health, University of Oxford, Oxford OX3 7LF, UK; 5 Million Veteran Program (MVP) Coordinating Center, Veterans Affairs Healthcare System, Boston, MA 02111, USA; 6 Department of Medical Epidemiology and Biostatistics, Karolinska Institutet, Stockholm 171 65, Sweden; 7 Pfizer Research and Development, Pfizer, Stockholm 113 63, Sweden; 8 Centre for Global Health Research, Usher Institute, University of Edinburgh, Edinburgh EH16 4UX, UK; 9 Department of Medical Epidemiology and Biostatistics, Karolinska Institutet, Stockholm 171 77, Sweden; 10 Estonian Genome Centre, Institute of Genomics, University of Tartu, Tartu 51010, Estonia; 11 Department of Genetic Medicine and Development, Faculty of Medicine, University of Geneva Medical School, Geneva 1211, Switzerland; 12 Division of Cardiovascular Medicine, Department of Medicine, Stanford University School of Medicine, Stanford, CA 90305-5406, USA; 13 School of Health and Social Studies, Dalarna University, Falun 79188, Sweden; 14 Division of Family Medicine and Primary Care, Department of Neurobiology, Care Sciences and Society (NVS), Karolinska Institutet, Stockholm 14183, Sweden; 15 Department of Epidemiology and Population Health, Stanford University School of Medicine, Stanford, CA 94305, USA; 16 Department of Medicine, Stanford University School of Medicine, Stanford, CA 94305, USA; 17 Berlin Institute of Health at Charité - Universitätsmedizin Berlin, Berlin 10178, Germany; 18 Institute of Medical Informatics, Charité - Universitätsmedizin Berlin, Freie Universität Berlin and Humboldt-Universität zu Berlin, Berlin 10115, Germany; 19 Center for Stroke Research (CSB), Charité - Universitätsmedizin Berlin, Freie Universität Berlin and Humboldt-Universität Berlin, Berlin 10117, Germany; 20 Department of Neurology with experimental Neurology, Charité - Universitätsmedizin Berlin, Freie Universität Berlin and Humboldt-Universität Berlin, Berlin 10117, Germany; 21 Institute of Biometry and Clinical Epidemiology, Charité - Universitätsmedizin Berlin, Freie Universität Berlin and Humboldt-Universität Berlin, Berlin 10117, Germany; 22 Department of Endocrinology and Metabolism, Charité - Universitätsmedizin Berlin, Freie Universität Berlin and Humboldt-Universität Berlin, Berlin 10117, Germany; 23 Department of Nephrology and Medical Intensive Care, Charité - Universitätsmedizin Berlin, Freie Universität Berlin and Humboldt-Universität Berlin, Berlin 13353, Germany; 24 Department of Cardiology, Angiology and Intensive Care Medicine, Campus Virchow Klinikum, Deutsches Herzzentrum der Charité(DHZC), Berlin 13353, Germany; 25 DZHK Partner Site Berlin, DZHK (German Centre for Cardiovascular Research), Berlin 10785, Germany; 26 Biobank Technology Platform, Max Delbrück Center for Molecular Medicine in the Helmholtz Association (MDC), Berlin 13125, Germany; 27 Genetics and Genomics of Cardiovascular Diseases, Max Delbrück Center for Molecular Medicine in the Helmholtz Association (MDC), Berlin 13125, Germany; 28 Hypertension-Caused End-Organ Damage, Max Delbrück Center for Molecular Medicine in the Helmholtz Association (MDC), Berlin 13125, Germany; 29 Integrative Vascular Biology, Max Delbrück Center for Molecular Medicine in the Helmholtz Association (MDC), Berlin 13125, Germany; 30 Division of Cardiology, Center for Molecular Medicine, Karolinska University Hospital, Solna, Stockholm, Sweden; 31 Department of Clinical Medicine, Aarhus University, Aarhus 8000, Denmark; 32 Department of Cardiology, Gødstrup Hospital, Herning 7400, Denmark; 33 British Heart Foundation Centre of Research Excellence, School of Clinical Medicine, Addenbrooke’s Hospital, University of Cambridge, Cambridge CB2 0BB, UK; 34 Department of Public Health and Primary Care, British Heart Foundation Cardiovascular Epidemiology Unit, University of Cambridge, Cambridge CB2 0BB, UK; 35 NIHR Blood and Transplant Research Unit in Donor Health and Behaviour, University of Cambridge, Cambridge CB2 0BB, UK; 36 Victor Phillip Dahdaleh Heart and Lung Research Institute, University of Cambridge, Cambridge CB2 0BB, UK; 37 Health Data Research UK, Wellcome Genome Campus, University of Cambridge, Hinxton CB10 1RQ, UK; 38 Nuffield Department of Population Health, University of Oxford, Oxford OX3 7LF, UK; 39 Division of Aging, Mass General Brigham, Harvard Medical School, Boston, MA 02130, USA; 40 Lothian Birth Cohorts, Department of Psychology, University of Edinburgh, Edinburgh EH8 9JZ, UK; 41 Department of Medical Epidemiology and Biostatistics, Karolinska Institutet, Stockholm 171 77, Sweden; 42 British Heart Foundation Centre of Research Excellence, School of Clinical Medicine, Addenbrooke’s Hospital, School of Clinical Medicine, Addenbrooke’s Hospital, Cambridge CB2 0BB, UK; 43 Public Health and Primary Care, Victor Phillip Dahdaleh Heart and Lung Research Institute, Cambridge CB2 0BB, UK; 44 Health Data Research UK Cambridge, Wellcome Genome Campus, University of Cambridge, Cambridge CB2 0BB, UK; 45 Department of Human Genetics, Wellcome Sanger Institute, Cambridge CB10 1SA, UK; 46 Nutrition and Dietetics, School of Health Science and Education, Harokopio University, Athens 17671, Greece; 47 Departement of Clinical Sciences in Malmö, Lund University, Malmö205 02, Sweden; 48 Uppsala Clinical Research Center, Uppsala University, Uppsala 751 85, Sweden; 49 Department of Medicine Solna, Karolinska Institutet, Stockholm 17177, Sweden; 50 Genomic Epidemiology Branch (GEM), International Agency for Research on Cancer (IARC), Lyon 69007, France; 51 Department of Clinical Sciences, Lund University, Helsingborg 25187, Sweden; 52 Department of Genetics, University Medical Center Groningen, Groningen 9713GZ, the Netherlands; 53 Department of Pediatrics, University Medical Center Groningen, Groningen 9713GZ, the Netherlands; 54 Department of Medicine, Brigham and Women’s Hospital, Boston, MA 02120, USA; 55 Department of Medicine, VA Boston Healthcare System, Boston, MA 02132, USA; 56 Department of Epidemiology, Erasmus MC University Medical Center Rotterdam, Rotterdam 3015 GD, the Netherlands; 57 German Center for Diabetes Research (DZD), Neuherberg 85764, Germany; 58 Institute of Epidemiology, Helmholtz Zentrum München, German Research Center for Environmental Health, Neuherberg 85764, Germany; 59 Research Unit of Molecular Epidemiology, Helmholtz Zentrum München, German Research Center for Environmental Health, Neuherberg 85764, Germany; 60 Institute of Translational Genomics, Helmholtz Zentrum München, German Research Center for Environmental Health, Neuherberg 85764, Germany; 61 Institute of Epidemiology, Research Unit of Molecular Epidemiology, Helmholtz Zentrum München, Neuherberg 85764, Germany; 62 School of Public Health, Imperial College London, London W12 0BZ, UK; 63 Department of Medical Sciences, Uppsala University, Uppsala 75185, Sweden; 64 Department of Psychiatry and Neurochemistry, Institute of Neuroscience and Physiology at the Sahlgrenska Academy, University of Gothenburg, Gothenburg 41346, Sweden; 65 Department of Medical Epidemiology and Biostatistics, Karolinska Institutet, Stockholm 171 77, Sweden; 66 Department of Oncology, Södersjukhuset, Stockholm 118 83, Sweden; 67 Clinical Memory Research Unit, Department of Clinical Sciences Malmö, Faculty of Medicine, Lund University, Lund 22100, Sweden; 68 Institute for Genetics and Cancer, University of Edinburgh, Edinburgh EH4 2XU, UK; 69 Partner Düsseldorf, German Center for Diabetes Research (DZD), Neuherberg 85764, Germany; 70 Institute for Clinical Diabetology, German Diabetes Center, Leibniz Center for Diabetes Research at Heinrich Heine University Düsseldorf, Düsseldorf 40225, Germany; 71 Division of Endocrinology and Diabetology, Medical Faculty, University Hospital Düsseldorf, Heinrich Heine University Düsseldorf, Düsseldorf 40225, Germany; 72 Department of Medical Sciences, Cardiology, Uppsala University, Uppsala 75185, Sweden; 73 Department of Psychology, University of Edinburgh, Edinburgh EH8 9JZ, UK; 74 Institute of Precision Medicine, The First Affiliated Hospital, Sun Yat-Sen University, Guangzhou 510080, China; 75 Department of Biostatistics, Boston University, Boston, MA 02215, USA; 76 Population Sciences Branch, National Heart Lung and Blood Institute, National Institute of Health, Framingham, MA 01702, USA; 77 Department of Laboratory Medicine, Institute of Biomedicine, Gothenburg 405 30, Sweden; 78 Clinical Genetics and Genomics, Sahlgrenska University Hospita, Gothenburg 413 45, Sweden; 79 Immunology, Genetics and Pathology, Uppsala University, Uppsala 74895, Sweden; 80 Institute of Neuroscience and Physiology, University of Gothenburg, Gothenburg 41345, Sweden; 81 Alnylam Pharmaceuticals, Inc., Cambridge, MA 02142, USA; 82 Cardiovascular Medicine, Stanford University, Stanford, CA 94305, USA; 83 MRC Human Genetics Unit, University of Edinburgh, Edinburgh EH4 2XU, UK; 84 Stanley Center for Psychiatric Research, Broad Institute of MIT and Harvard, Cambridge, MA 02142, USA; 85 Department of Psychiatry and Psychotherapy, Charité– Universitätsmedizin Berlin, Berlin 10117, Germany; 86 German Center for Mental Health (DZPG), Partner Site Berlin/Potsdam, Berlin 10117, Germany; 87 Institute of Neuroscience and Physiology, University of Gothenburg, Gothenburg 413 45, Sweden; 88 Population Sciences Branch, National Heart, Lung, and Blood Institute of the National Institutes of Health, Framingham, MA 02461, USA; 89 Framingham Heart Study, Framingham, MA 01702, USA; 90 School of Public Health, Peking University, Beijing 100191, China; 91 Department of Medicine, Vanderbilt University Medical Center, Nashville, TN 37213, USA; 92 Clinical Memory Research Unit, Department of Clinical Sciences Malmö, Lund University, Malmö20213, Sweden; 93 Department of Clinical Science and Education, Karolinska Institutet, Stockholm 118 83, Sweden; 94 Core Unit Proteomics, Berlin Institute of Health at Charité - Universitätsmedizin Berlin, Berlin 13125, Germany; 95 Proteomics Platform, Max Delbrück Center for Molecular Medicine, Berlin 13125, Germany; 96 Medical Epidemiology, Department of Surgical Sciences, Uppsala University, Uppsala 751 85, Sweden; 97 Department of Health Science and Technology, Aalborg University, Gistrup 9260, Denmark; 98 Clinical Trial Service Unit, Epidemiological Studies Unit (CTSU), Nuffield Department of Population Health, University of Oxford, Oxford OX3 7LF, UK; 99 Department of Congenital Disorders, Statens Serum Institute, Copenhagen 2300, Denmark; 100 Department of Diabetes, Endocrinology & Reproductive Biology, University of Dundee, Dundee DD1 9SY, UK; 101 Department of Immunology and Inflammation, Imperial College London, London W120HS, UK; 102 Robertson Centre for Biostatistics, School of Health and Wellbeing, College of Medical, Veterinary and Life Sciences, University of Glasgow, Glasgow G128TB, UK; 103 Department of Public Health, University of Split, School of Medicine, Split 21000, Croatia; 104 British Heart Foundation Cardiovascular Epidemiology Unit, Department of Public Health and Primary Care, University of Cambridge, Cambridge CB2 0BB, UK; 105 German Center for Mental Health, DZPG, site Berlin-Potsdam, Berlin 10117, Germany; 106 Partner Düsseldorf, German Center for Diabetes Research (DZD), Neuherberg 80992, Germany; 107 Institute of Clinical Diabetology, German Diabetes Center, Düsseldorf 40225, Germany; 108 Department of Endocrinology and Diabetology, Heinrich Heine University, Düsseldorf 40225, Germany; 109 Novo Nordisk Research Centre Oxford, Novo Nordisk, Oxford OX3 7FZ, UK; 110 SCALLOP Consortium; 111 Greater Bay Area Institute of Precision Medicine, State Key Laboratory of Genetic Engineering, Center for Evolutionary Biology, School of Life Sciences, Fudan University, Shanghai 200032, China; 112 Science for Life Laboratory, Department of Protein Science, KTH Royal Institute of Technology, Solna 171 65, Sweden; 113 Department of Medical Sciences, Clinical Chemistry, Uppsala University, Uppsala 75185, Sweden; 114 Department of Molecular and Clinical Medicine, Institute of Medicine, Gothenburg University, Gothenburg 413 34, Sweden; 115 Science for Life Laboratory, Gothenburg University, Gothenburg 413 34, Sweden; 116 Department of Cardiology, Clinical Sciences, Lund University, Lund 221 84, Sweden; 117 Wallenberg Center for Molecular Medicine, Lund University Diabetes Center, Lund University, Lund 221 84, Sweden; 118 Department of Cardiology, Sahlgrenska University Hospital, Gothenburg 413 34, Sweden; 119 Department of Heart Failure and Valvular Disease, Skåne University Hospital, Lund 221 85, Sweden; 120 Department of Clinical Genetics and Genomics, Sahlgrenska University Hospital, Gothenburg 413 90, Sweden; 121 Institute of Biomedicine, Department of Laboratory Medicine, the Sahlgrenska Academy, University of Gothenburg, Gothenburg 405 30, Sweden; 122 Englander Institute for Precision Medicine, Weill Cornell Medicine, New York, NY 10021, USA; 123 Bioinformatics Core, Weill Cornell Medicine-Qatar, Doha 24144, Qatar; 124 The George Institute for Global Health, University of New South Wales, Sydney, NSW 2031, Australia; 125 Partner Munich-Neuherberg, German Center for Diabetes Research (DZD), Neuherberg 85764, Germany; 126 Institute for Medical Information Processing, Biometry and Epidemiology - IBE, Faculty of Medicine, Ludwig-Maximilians-Universität in Munich, Munich 81377, Germany; 127 Genomics Core Facility, Department of Internal Medicine, Erasmus Medical Center, Rotterdam, 3015 GD, the Netherlands; 128 Department of Orthopedics and Sports Medicine, Erasmus Medical Center, Rotterdam 3000DR, the Netherlands; 129 Population Health and Genomics, University of Dundee, Dundee DD19SY, UK; 130 Department Medical Sciences, Uppsala University, Uppsala 75285, Sweden; 131 Uppsala Clinical Research Center, Uppsala University, Uppsala 75185, Sweden; 132 BeLOVE Unit, Berlin Institute of Health at Charité - Universitätsmedizin Berlin, Berlin 10178, Germany; 133 Center for Stroke Research (CSB), Charité - Universitätsmedizin Berlin, Freie Universität Berlin and Humboldt-Universität Berlin, Berlin 12203, Germany; 134 Department of Neurology, Charité - Universitätsmedizin Berlin, Freie Universität Berlin and Humboldt-Universität Berlin, Berlin 12203, Germany; 135 Department of Gastroenterology and Hepatology, University Medical Center Groningen, University of Groningen, Groningen 9700RB, the Netherlands; 136 National Research Council, Institute of Genetic and Biomedical Research, Cagliari 09042, Italy; 137 Department of Medicine, Stanford University School of Medicine, Palo Alto, CA 94305, USA; 138 TUM School of Medicine and Health, Technical University of Munich (TUM), TUM University Hospital, Munich 81675, Germany; 139 Department of Public Health and Primary Care, British Heart Foundation Cardiovascular Epidemiology Unit, University of Cambridge, Cambridge CB1 8RN, UK; 140 Victor Phillip Dahdaleh Heart & Lung Research Institute, University of Cambridge, Cambridge CB2 0BB, UK; 141 Department of Genetics, University of Groningen, Medical Center Groningen, Groningen 9700 RB, the Netherlands; 142 Proteomics Platform, Max-Delbrück-Center for Molecular Medicine in the Helmholtz Association (MDC), Berlin 13125, Germany; 143 Chinese Academy of Medical Sciences Oxford Institute, Nuffield Department of Medicine, University of Oxford, Oxford OX3 7BN, UK; 144 Target Discovery Institute, Nuffield Department of Medicine, University of Oxford, Oxford OX3 7FZ, UK; 145 Department of Aging, Brigham and Women’s Hospital, Boston, MA 02215, USA; 146 Department of Medicine, Harvard Medical School, Boston, MA 02215, USA; 147 Department of Medical Epidemiology and Biostatistics, Karolinska Institutet, Stockholm 17176, Sweden; 148 Max Planck Institute for Molecular Genetics, Berlin 14195, Germany; 149 These authors contributed equally; 150 These authors contributed equally; 151 Lead contact

## Abstract

Understanding the genetic regulation of circulating protein levels can provide new insights into disease mechanisms. Here, we present the largest proteogenomic study to date (*n* = 78,664 participants across 38 studies), identifying >24,000 protein quantitative trait loci (QTLs) associated with 1,116 proteins, acting near to (*n* = 5,040) or distant (*n* = 19,698) from the cognate gene. Using machine learning-guided effector gene assignment, we provide genetic evidence for pathways, cell types, and tissues that modulate circulating protein levels, highlighting N-linked glycosylation as an important regulatory pathway. We demonstrate that genetic instruments of protein production/function (“*cis*”) versus modulation (“*trans*”) reveal distinct phenotypic insights. We identify proteins as candidates for drug targets and engagement (e.g., plasma furin and cardiovascular diseases) by comparing *cis*-based genetic evidence with protein-disease associations. Systematic triangulation of *trans*-protein QTLs (pQTLs) with genetic and protein associations across many diseases highlights potential drug repurposing opportunities, e.g., tyrosine kinase 2 (TYK2) inhibitors for rheumatoid arthritis. Our multi-cohort meta-analyses generate proteogenomic insights into disease mechanisms and new treatment opportunities.

## In brief

A large-scale multi-cohort proteogenomic study identifies genetic loci influencing circulating protein levels, revealing pathways and cell types that regulate the circulating proteome and highlighting disease insights and evidence for potential therapeutic opportunities.

## Graphical Abstract

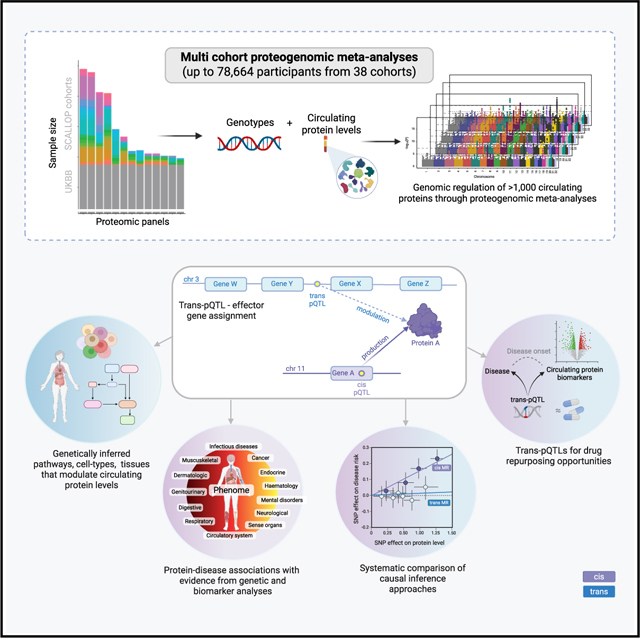

## INTRODUCTION

Most disease-predisposing variation in the human genome resides in non-coding regions, limiting inference about causal genes and mechanisms.^1,2^ Systematic functional characterization of disease-associated variants can guide the identification of clinically relevant mechanisms,^3–5^ but model systems are difficult to scale or translate directly to human biology.^1–3^ High-throughput, broad-coverage proteomic technologies that simultaneously target thousands of proteins in human blood can help to link non-coding variants to disease-relevant mechanisms via proteins.^6–24^ Although such proteogenomic studies have highlighted novel clinically relevant mechanisms and potential drug targets or indications,^6–24^ important limitations remain. First, broad-coverage studies to date have almost exclusively relied on proximally acting *cis*-variants (i.e., *cis*-protein quantitative trait loci [*cis*-pQTLs]) to derive disease insights. However, non-coding variants are likely to map to regulatory regions directly affecting multiple genes encoded close by (as also demonstrated for gene expression QTLs^25^) or indirectly regulate proteins produced by genes that are elsewhere in the genome. Second, a better understanding of the polygenic architecture affecting diagnostic, predictive, or prognostic protein biomarkers can be important, as recently demonstrated for prostate-specific antigen and prostate cancer prediction.^26^ Finally, robust and generalizable identification of pQTLs requires replication across diverse populations, which until now has rarely been pursued for broad-coverage proteomics.^18,23,24^

Here, we present multi-cohort proteogenomic meta-analyses of up to 1,161 protein targets in up to 78,664 individuals across 38 international cohorts (see STAR Methods and Figure S1A) to address these limitations and provide a conceptual advance by integrating and improving the biological interpretability of *trans*-pQTL effects, i.e., pQTLs that are not nearby the protein-encoding gene for the protein they associate with. We identify pathways, such as N-linked glycosylation, tissues, and cell types that regulate the plasma proteome based on human genetic evidence and demonstrate divergence of findings from *cis*- versus *trans*-focused genetic analyses across diseases. Systematically comparing *cis*-based gene-to-disease versus measured protein-to-disease associations in up to 1.3 million participants, we demonstrate limited convergence, with only a few examples supported by evidence from both approaches. We finally demonstrate the value of the many newly identified pleiotropic (*trans*-)pQTLs by illustrating how they can inform disease mechanisms, including druggable opportunities.

## RESULTS

### Multi-cohort genome-proteome-wide pQTL discovery

We performed a proteogenomic meta-analysis of 1,161 protein targets measured in blood across 38 cohorts, including up to 78,664 participants of European ancestry (Table S1A; for study design see Figure S1). We identified 14,690 regional sentinel variants (*n* = 1,009 *cis*-pQTLs, *n* = 13,681 *trans*-pQTLs) for 1,116 protein targets at a Bonferroni-corrected significance threshold. We observed at least one pQTL close to the cognate protein-coding gene (i.e., *cis*-pQTL) or elsewhere in the genome (i.e., *trans*-pQTL) for 87.1% and 94.1% of the tested protein targets (*n* = 1,161), respectively.

**Figure 1.**
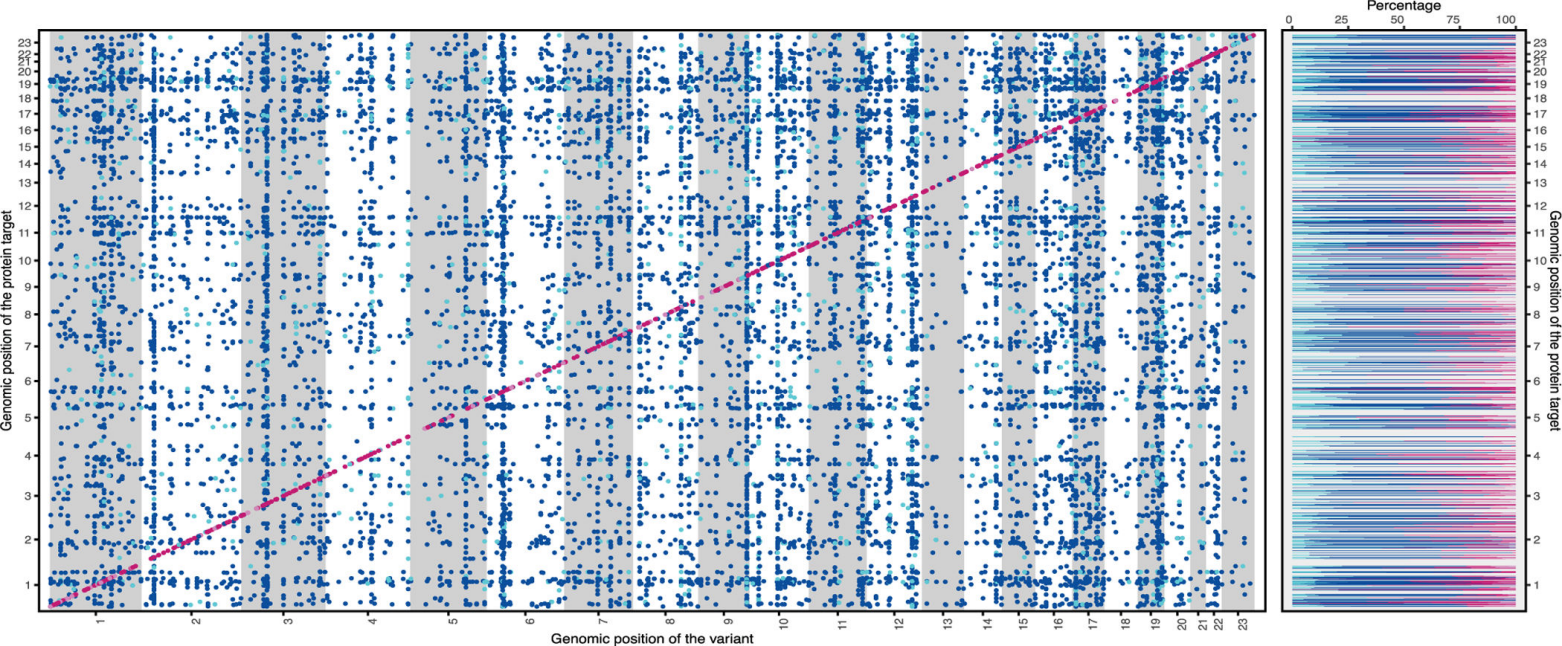
Fine-mapped protein quantitative trait loci in SCALLOP and UKBB meta-analyses The left panel displays the genomic coordinates of the genetic variants and protein-encoding genes on the *x* and *y* axes, respectively. The pQTLs were colored by their category (dark pink, high-confidence *cis*-pQTLs; light pink, low-confidence *cis*-pQTLs; dark blue, high-confidence *trans*-pQTLs; light blue, low-confidence *trans*-pQTLs). The right panel displays the proportion of pQTLs in each “confidence” category (see STAR Methods) for each protein target. Relevant summary statistics can be found in Table S2. See also Figures S1, S2, and S3.

Bayesian fine-mapping^27^ of the 14,690 identified genomic regions revealed 24,738 independent sets of credible variants associated with 1,116 protein targets (*n* = 5,040 *cis*-pQTLs and *n* = 19,698 *trans*-pQTLs; Figure 1). We observed a median of 4 credible sets (interquartile range [IQR]: 2–7; Table S2), including independent genetic variants within/nearby cognate protein-coding genes. We observed considerable correlation (r = 0.6) of effect sizes for the identified regional sentinel variants in cohorts of non-European ancestry (*n* = 3 cohorts), where overlapping information was available (3,709 pQTLs for 501 protein targets; Figure S2).

For about a third of the protein targets with at least one *cis-*pQTL, we observed evidence that at least one of the *cis* genetic signals was also colocalized with expression QTLs of the protein-coding gene in one or more of 43 tissues, indicating these as potential origins for given blood proteins^25^ (see STAR Methods and Table S3). Functional annotation of identified pQTLs further replicated the previous observation of high rates of functional, e.g., missense, variants among *cis*-pQTLs (10.6%), although we observed similarly high rates of functional variants among *trans*-pQTLs (10.4%), including those associated with many protein targets. The latter may imply that broad effects on the plasma proteome require disturbance of causative gene products rather than subtle effects on gene regulation, as has been seen for most genome-wide association study (GWAS) traits.

Most fine-mapped pQTLs were “high confidence” (82.3% of the 5,040 *cis*- and 83.3% of the 19,698 *trans*-fine-mapped pQTLs), defined as (1) genome-wide significant (*p* < 5 × 10^−8^); (2) directionally consistent in the overall meta-analysis between SCALLOP cohorts and UK Biobank (UKBB) for *cis*- and *trans-*pQTLs; and, additionally, with (3) no or minimal heterogeneity (*p*_het_ > 1 × 10^−4^) for *trans*-pQTLs (Figure 1; Table S2). Of those, 278 *cis*- and 4,013 *trans*-pQTLs or their proxies (r^2^ > 0.1) have not previously been reported for the same protein target^6–24^ (Table S2). Investigating the impact of cohort-level characteristics from SCALLOP cohorts (mean cohort age, mean cohort BMI, composition of sex in the cohort, whether any cases were included in the cohort, fasting status, and blood-based sample type), we observed that the heterogeneity was mostly explained by small variations in very strong genetic effects (Tables S1 and S4).

We observed large variation among protein targets when contrasting variance explained (mean = 8.4%, IQR = 4.9%–10.4%) by loci identified in this study and the estimated remaining polygenic background (mean = 10.2%, IQR = 6.6%–13.6%; Figure S3A). Extreme examples ranged from monogenic proteins such as Fc receptor-like protein 3 (FCRL3, variance explained = 45.3%; polygenic background = 3.8%), mainly explained by identified *cis* loci, to polygenic ones with contributions from *cis* and *trans* loci, as well as the polygenic background, such as vascular endothelial growth factor receptor 2 (VEGFR-2 encoded by *KDR*, variance explained = 21.6%; polygenic background = 16.4%). pQTLs close to the protein-coding gene explained, on average, (i.e., *cis*), more variation in plasma protein levels compared with the cumulative impact of variation elsewhere in the genome (i.e., *trans*) (Figure S3A; Table S5). However, extreme outliers included intercellular adhesion molecule 2 (ICAM2), for which *cis*-pQTLs explained only 0.3% of variance compared with 52.7% explained by *trans*-pQTLs or alpha-L-fucosidase 1 (FUCA1; 6.3% by *cis*-pQTLs versus 68.4% by *trans*-pQTLs). We further observed 261 protein targets for which the linear dependency between explained variance by pQTLs and polygenic heritability estimates did not persist, providing evidence that our study may have already saturated pQTL discovery for these proteins (Figure S3B).

### Understanding characteristics of protein targets under genetic regulation

**Figure 2.**
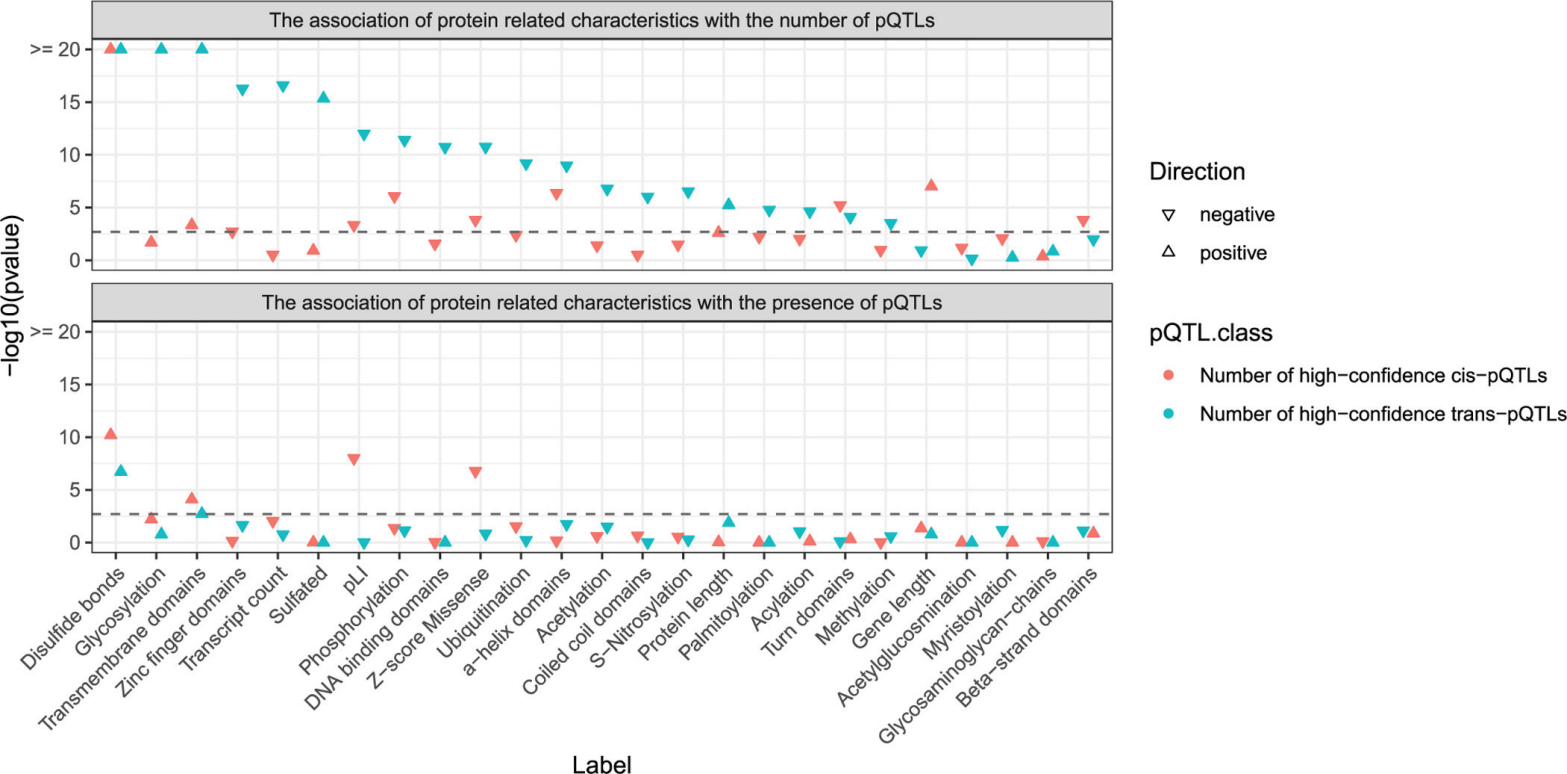
Protein characteristics associated with the presence and number of pQTLs based on zero-inflated Poisson regression models Results for high-confidence *cis*- and *trans*-pQTLs are colored red and blue, respectively. The results from Poisson regression (i.e., association with the number of pQTLs with protein characteristics) and logistic regression (i.e., association with presence or lack of pQTLs with protein characteristics) are represented in the top and bottom panels, respectively. The horizontal dashed line represents the Bonferroni-corrected significance threshold (*p* < 2 × 10^−3^). The *p* value display was capped at *p* < 1 × 10^−20^ for display purposes; *p* values can be found in Table S5. Abbreviations: pLI, probability of loss-of-function intolerance.

A higher number of pQTLs among protein targets showed a significant association with the presence of disulfide bonds or a transmembrane domain (Figure 2; Table S6), likely explained by measuring those in the most relevant compartment (i.e., the circulation). In contrast, a higher constraint of the protein-coding gene (i.e., probability of loss-of-function intolerance [pLI]) was inversely associated with the presence and number of *cis*-pQTLs (*p* = 4.8 × 10^−4^) as well as the number of *trans*-pQTLs (*p* = 9.9 × 10^−13^).

Proteins with a higher number of *trans*-pQTLs were further characterized by those enriched for characteristics of secreted proteins,^28^ such as glycosylation and sulfation, but depleted for structural features of intracellular proteins, such as zinc-finger and DNA-binding domains (Figure 2; Table S6). We note that these findings persisted upon controlling for missingness rates for a given protein; that is, our findings are unlikely to be driven by higher precision for proteins reliably measured with the assay technology (Table S6).

### Effector genes at *trans*-pQTLs inform pathway and cell-type contributions

Most identified genetic regulation of the human proteome does not occur within or nearby a protein’s cognate gene but rather elsewhere in the genome. Mapping effector genes in distal genomic regions to pQTLs that associate with plasma protein levels has been challenging but can benefit from prior biological knowledge, for example, in ligand-receptor pairs.^20^

We identified at least one candidate effector gene with at least medium confidence (candidate gene score > 1 out of 3) for more than half of the *trans*-pQTLs (*n* = 11,261) by incorporating prior biological knowledge in a machine learning framework (see STAR Methods and Table S7). This included 1,534 high-confidence assignments with a candidate gene score ≥2 (maximum: 2.704 out of 3). For two-thirds of the loci (*n* = 13,881), the distribution of candidate gene scores across genes indicated a single causal gene as the most likely. As a partial external validation, we observed 552 *trans*-pQTLs mapping to effector genes that encoded high-confidence protein-protein interaction partners from the STRING network, which was not part of our gene annotation pipeline.

**Figure 3.**
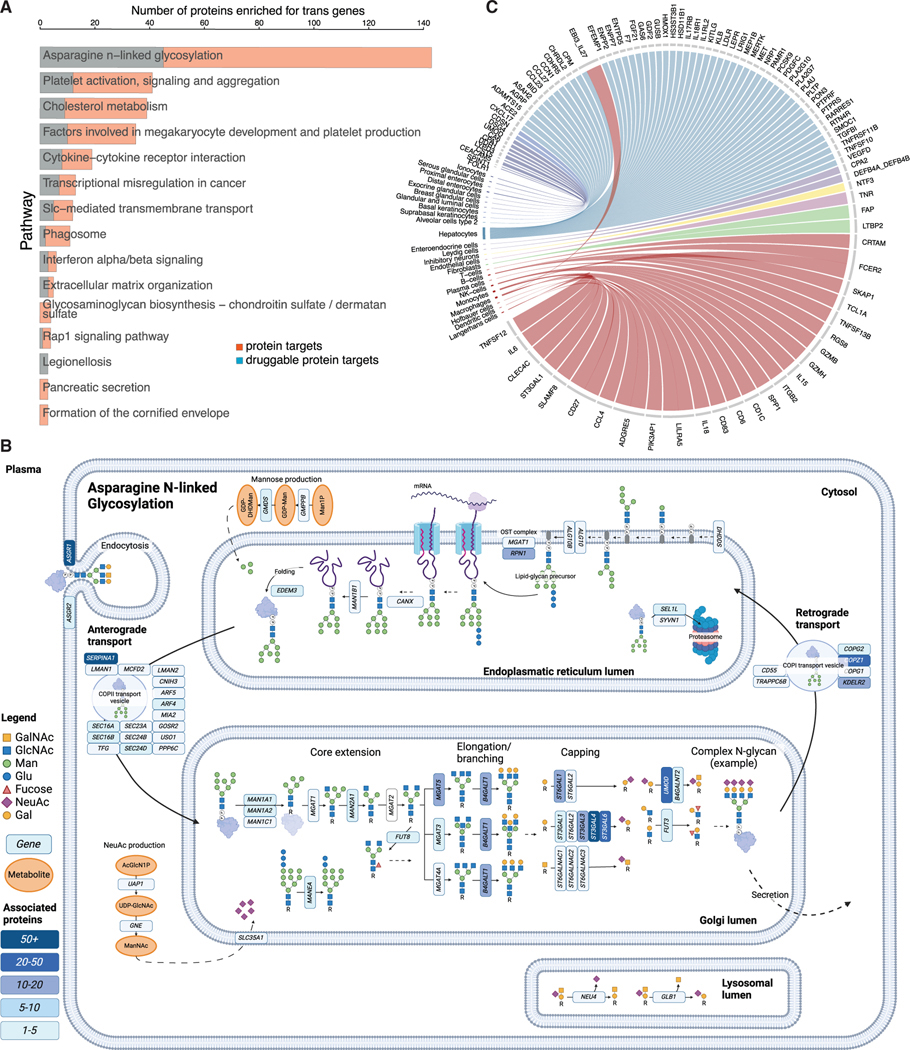
Effector gene assignment at *trans*-pQTLs identifies major pathways and cell types involved in plasma protein modulation (A) Pathways most frequently enriched among top-prioritized *trans*-effector genes across all 439 protein targets with at least one significantly enriched (FDR < 5%) pathway. Proteins that are the targets of approved drugs or those in clinical development are highlighted in gray. (B) Pathway diagram showing the role of *trans*-pQTL effector genes in “asparagine N-linked glycosylation” based on the R-HSA-446203 REACTOME pathway. Only members of the pathway that were assigned at least one *trans*-pQTL are displayed to retain readability, and colors indicate how often the gene was assigned as an effector gene across protein targets. GalNAc, N-acetyl-D-galactosamine; GlcNAc, N-acetylglucosamine; Man, mannose; Glu, glucose; NeuAc, N-acetylneuraminic acid; Gal, galactose; created in https://BioRender.com. (C) Chord diagram illustrating significant (FDR < 5%) enriched cell types among *trans*-effector genes across assayed protein targets. Colors are grouped by the Human Protein Atlas categories. Relevant summary statistics can be found in Table S8.

We next sought to identify the pathways, cell types, and tissues involved in the *trans* regulation of the assayed protein targets. We identified at least one significantly enriched pathway among the top-prioritized *trans*-effector genes for 431 protein targets (Table S8A). Those segregated roughly into two categories. For 101 protein targets, effector genes were enriched for at least one pathway that also involved the *cis*-protein, providing evidence that higher/lower circulating protein levels were, at least partly, a result of generally higher/lower pathway activity. For example, *trans*-effector genes for the low-density lipoprotein (LDL)-receptor were more than 85-fold enriched for members of cholesterol metabolism (false discovery rate [FDR] = 9.8 × 10^−15^). In contrast, for most assayed protein targets (*n* = 330), pathways enriched among *trans*-effector genes rather pointed to distal, systemic regulators of (circulating) protein levels. These included pathways related to post-translational modifications, such as “asparagine N-linked glycosylation” (*n* = 143 protein targets; Figure 3B), as well as pathways related to platelet and blood cell biology more broadly, such as platelet aggregation (*n* = 41) (Figure 3A). N-glycosylation is the most common post-translational modification of secreted proteins, determining correct folding and secretion, as well as affecting half-life and signaling properties of protein targets.^29^ We note that the most pleiotropic *trans*-effector genes mapped to elongation, branching, and capping of the glycans, modulating stability rather than earlier processes, such as folding in the endoplasmic reticulum.

We identified 95 and 97 protein targets for which *trans*-effector genes were significantly (FDR < 5%) enriched for the specific/enhanced expression of the protein-coding gene in one of 12 tissues and 29 cell types, respectively (Figure 3C; Tables S8B and S8C). This included 44 protein-tissue and 76 protein-cell-type pairs that were not the primary site of protein production of the *cis*-protein, potentially demonstrating cross-organ communication in protein homeostasis. Most prominently, effector genes with enhanced expression in the liver, specifically hepatocytes, were linked to many circulating protein targets. Effector genes expressed in immune cell populations that modulate interleukins (ILs), e.g., genes with enhanced expression in natural killer (NK) cells, were also more than 13-fold enriched (FDR = 3.4 × 10^−6^) for *trans*-effector genes of the NK cell activator IL-15. Other examples included the brain-specific extracellular matrix protein tenascin R, for which *trans*-effector genes were more than 20-fold enriched (FDR = 2.8 × 10^−8^) for genes with enhanced expression in endothelial cells, or neurotrophin 3, a neuronal growth factor, with *trans*-effector genes being strongly enriched for enhanced expression in alveolar type 2 cells in the lung. We also observed examples with *trans*-effector genes being enriched for multiple, different cell types for the same protein target. For example, *trans*-effector genes for IL-27 with enhanced expression in hepatocytes and Kupffer cells (resident macrophages in the liver) were linked to glycosylation and recognition of pathogens, respectively.

### Molecular versus phenome-wide pleiotropy

**Figure 4.**
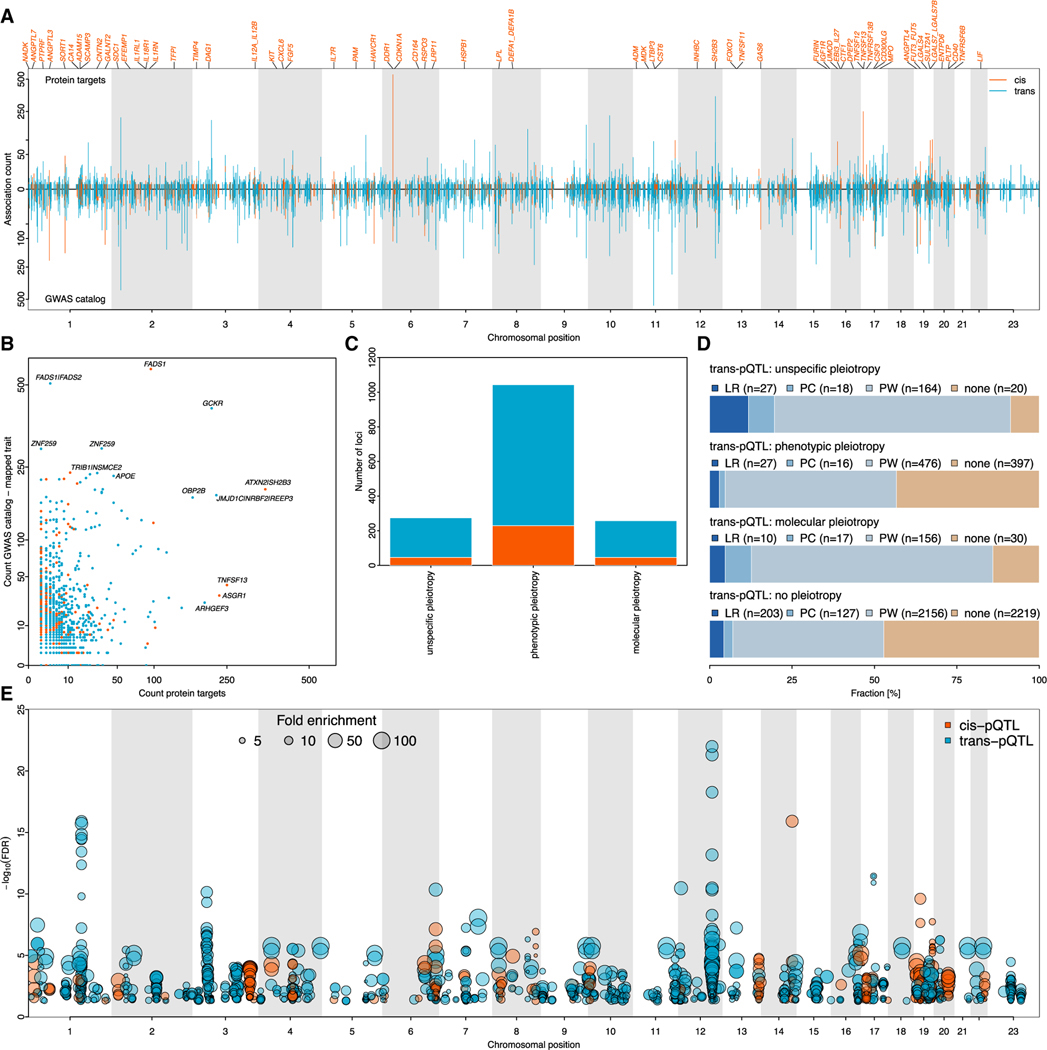
Molecular versus phenome-wide pleiotropy (A) Counts of associated protein targets (upper) and associated phenotypes (lower) across 10,461 independent loci associated in the presented study. Phenotype associations were obtained from the GWAS catalog (including proxies) but by collapsing several studies of the same trait using the “mapped trait” column. Genetic variants acting as *cis*-pQTLs are shown in orange and otherwise in blue. Genes for *cis*-pQTLs associated with 10 or more phenotype categories are assigned on top of the plot. The *y* axis has been square-root transformed. (B) Scatterplot opposing the counts from (A), with most pleiotropic effector genes for each category being annotated. (C) Bar plot showing the number of pQTLs with evidence for different patterns of pleiotropy. (D) Fraction of candidate effector genes for *trans*-pQTLs according to biological categories in relation to associated protein targets. LR, ligand-receptor pair; PC, protein complex; PW, pathway gene. (E) Summary of pathway enrichment among pleiotropic pQTLs that have been reported at least once in the GWAS catalog. Dots indicate significantly enriched pathways (FDR > 5%). The size of the dot is proportional to the fold enrichment. Position along the *x* axis indicates the genomic location of the pQTL.

Almost half (43.4%; *n* = 4,547) of all independent pQTLs we identified have been reported to associate with at least one non-proteomic trait, e.g., disease susceptibility, in the GWAS catalog^30^ (see STAR Methods and Figures 4A–4C). This included a >4-fold enrichment (odds ratio [OR]: 4.11; *p* < 1.1 × 10^−230^) of *trans*- compared with *cis*-pQTLs, demonstrating the importance of understanding the polygenic architecture of plasma proteins (Figure 4; Table S9A). We subsequently characterized the pleiotropic genetic variants into three categories: (1) “molecular pleiotropy” (associated with >5 proteins and ≤5 non-proteomic phenotypes), (2) “phenotypic pleiotropy” (associated with >5 non-proteomic phenotypes and ≤5 proteins), and (3) “unspecific pleiotropy” (associated with >5 proteins and >5 non-proteomic phenotypes) (Figures 4B and 4C).

More than half (332 out of 533) of the pQTLs that were pleiotropic at the protein level also showed phenotypic pleiotropy in the GWAS catalog. Associated effector genes were >2-fold enriched for enhanced expression in hepatocytes (OR = 2.82; FDR = 3.8 × 10^−6^), aligning with the liver’s role in whole-body homeostasis. This included 229 *trans*-pQTLs consistently enriched to likely act on protein complexes (OR = 1.83; *p* < 2.7 × 10^−2^), ligand-receptor pairs (OR = 2.73; *p* < 3.8 × 10^−5^), or pathway partners (OR = 7.66; *p* < 3.5 × 10^−13^) of associated protein targets compared with other modes of pleiotropy (Figure 4D), illustrating that part of the plasma proteome may be a surrogate of critical disease-predisposing mechanisms in tissues. For example, we identified rs10849448 as a *trans*-pQTL for eight protein targets, likely acting via lymphotoxin beta receptor (*LTBR*) (score 1.8), which encodes for LTBR, the receptor for lymphotoxin (LTA), which was among the associated protein targets. The same variant, or proxies thereof (r^2^ > 0.8), has been reported to associate with >20 traits in the GWAS catalog, including celiac disease, chronic obstructive pulmonary disease, primary biliary cirrhosis, and different measures of lymphocytes. Although LTBR is not expressed on lymphocytes, signaling via LTBR is essential for the development of tertiary lymphoid structures that are associated with chronic inflammatory diseases.^31^ Pharmacological blocking of LTBR signaling has further been shown to revert airway fibrosis in mouse models of smoking-induced chronic obstructive pulmonary disease.^32^

In general, genetic variants associated with multiple proteins might guide the interpretation of otherwise cryptic GWAS loci. We identified 285 pQTLs previously reported for non-proteomic traits in the GWAS catalog and among which associated proteins were significantly (FDR < 0.05) enriched for one or more pathways (Figure 4E; Table S9B). For example, we observed that two independent *trans*-pQTLs were 38-fold enriched (FDR < 5.5 × 10^−4^) for proteins associated with keratinization and had been previously linked to the risk of acne. Although one of the putative effector genes, *ERRFI1*, had been linked to skin morphogenesis,^33^ the role of the second one, *SEMA4B*, has so far been elusive,^34^ and we provide evidence for a potential function in skin morphogenesis or homeostasis.

### Contrasting proximal versus distal genetic regulation of plasma proteins reveals discordant phenotypic consequences

Successfully integrating proximal, i.e., *cis*-based, genetic regulation of plasma protein levels with disease risk variants has a high biological prior that the protein is involved in disease onset. We found evidence for 300 such protein-disease pairs, combining our results with >700 diseases from the FinnGen project^35^ through causal inference methods for disease follow-up. However, only 73 examples were supported by both a dose-response relationship, via Mendelian randomization (MR), and statistical colocalization of genetic risk signals, emphasizing the need for complementary evidence for the genetic prioritization of candidate causal genes underlying diseases (Table S10A).

**Figure 5.**
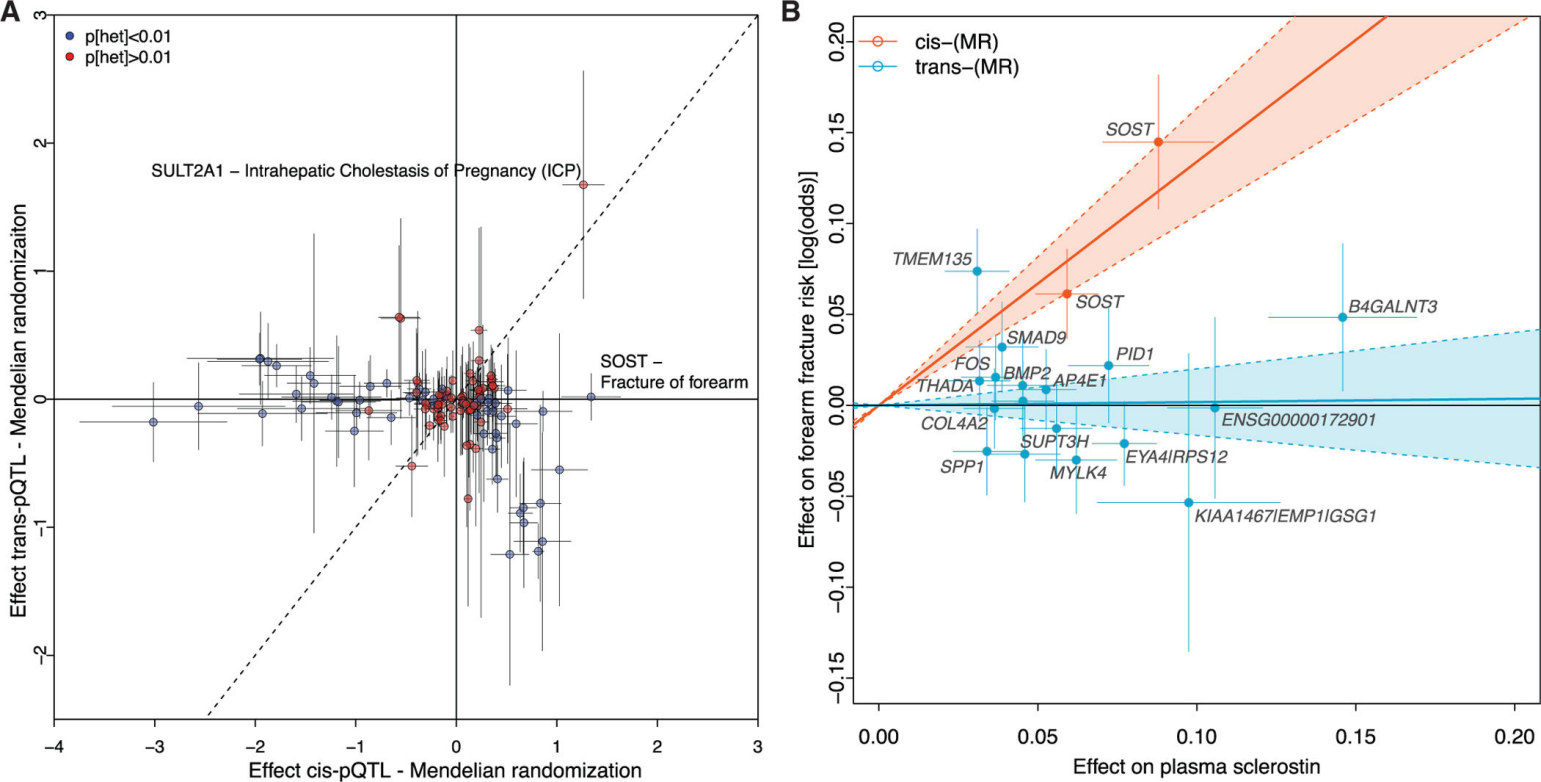
Effects of utilizing proximal (*cis*) and distal (*trans*) genetic variants to link proteins to disease risk (A) Comparison of effect estimates from MR analyses using only *cis*-pQTLs (*x* axis) versus *trans*-pQTLs (*y* axis) for high-confidence protein-disease links based on *cis* findings. Colors indicate evidence for effect heterogeneity between estimates derived from *cis*- and *trans-pQTLs*. (B) Causal effect estimates and 95% confidence intervals (CIs) using *cis*- (orange) and *trans*-pQTLs for plasma SOST levels on fracture risk. Relevant summary statistics can be found in Table S10.

For one-third of the *cis*-based examples (*n* = 115 out of 300), we identified enough protein-specific variants acting in *trans* to systematically test for independent support by MR. We observed supporting evidence for a third (*n* = 31) of *cis*-based protein-disease examples by demonstrating a directionally consistent association when using only *trans*-pQTLs for causal inference (*p*_het_ > 0.01; Figure 5; Table S10B). This included proteins with known roles in blood, such as known pharmacological targets that act at the blood-tissue interface (e.g., proprotein convertase subtilisin/kexin type 9 [PCSK9] and coronary artery disease). However, we found no evidence for an enrichment of proteins specific to blood cells (e.g., lymphoid tissue: OR = 1.30; *p* = 0.73) or being actively secreted into blood (OR = 0.78; *p* = 0.56) among those with consistent effects in *cis* and *trans*. Proteins likely conveying risk within tissues included sulfotransferase family 2A member 1 (SULT2A1), linked to intrahepatic cholelithiasis of pregnancy (ICP) (Figure 5). Genetic variation near *SULT2A1*, which encodes a sulfotransferase with a role in the metabolism of endogenous compounds and drugs, has previously been shown to increase the risk for ICP,^36^ and gallstones in general,^37^ mainly attributed to increased hepatic expression and hence activity of the enzyme to produce supersaturated bile that promotes gallstone formation.^16^ We provide evidence that potential distal regulators of SULT2A1 abundance, e.g., via the *trans*-pQTL rs16919533, which we assigned to *PANX1* with moderate confidence (gene score: 1.0), may also contribute to disease risk.

However, we also observed significant differences (*p*_het_ < 0.01) between results from well-powered *trans*-pQTL MRs not supporting (*n* = 41), or even opposing (*n* = 14), robust *cis*-pQTL evidence (Figure 5). For example, the well-documented effect of changes in sclerostin (SOST), a negative regulator of bone formation, on fracture risk observed with *cis*-pQTLs (beta_*cis*_ = 1.34, *p*_*cis*_ < 5.6 × 10^−19^) was null (*beta*_*trans*_ = 0.02, *p*_*trans*_ = 0.84) when considering 16 specific *trans*-pQTLs that cumulatively explained a higher amount of variation in plasma SOST levels (Figure 5). We observed a similar null effect, when further excluding bone mineral density-associated *trans*-pQTLs in an additional sensitivity analysis.^38^

In general, naive incorporation of *trans*-pQTLs into MR-based causal inference resulted in even larger disagreement with <20% of findings having concordant support based on *cis*-instruments, likely driven by pleiotropy not captured by statistical techniques (15 out of 96 for *cis*-/*trans*-pQTL-based MR and 6 out of 230 for *trans*-pQTL-based MR; Table S10C). The few potentially true positive findings missed by our biologically informed approach included plasma levels of von Willebrand factor and von Willebrand disease (*cis*/*trans*-pQTL MR: beta = −3.42, *p* < 2.9 × 10^−16^).

### Limited concordance between protein-phenotype associations identified using *cis*-focused genetic and observational studies

Blood proteins with a causal role in disease development are attractive targets for drug development and as markers of target engagement. We therefore systematically triangulated prevalent and incident plasma protein biomarker studies in up to 52,164 UKBB participants (using logistic regression and survival analysis) with high-confidence genetic inference in a pan-biobank effort, including up to 1,296,701 participants (using *cis*-MR and colocalization) for 517 diseases (Table S11).

**Figure 6.**
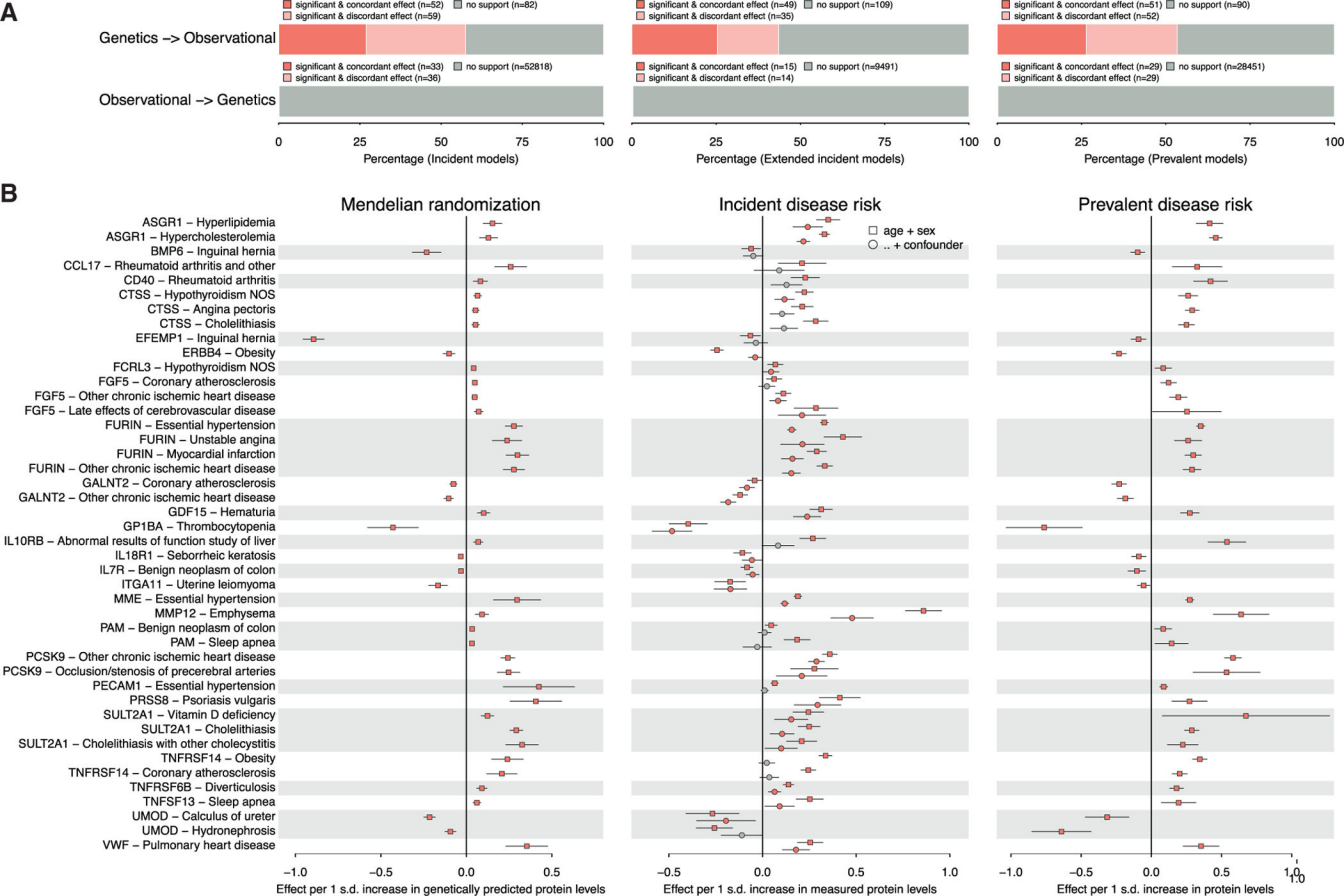
Concordance of genetically informed and observational protein biomarker studies (A) Bar charts illustrating the fraction of protein-disease pairs with support from independent biomarker prioritization strategies. The upper panel illustrates starting with evidence from MR and colocalization using *cis*-pQTLs. The bottom panel illustrates starting with evidence from observational studies using survival analysis (left and middle) or prevalent disease status (right). (B) Protein-disease examples with concordant support from genetic and (prospective) observational studies, illustrating effect estimates with 95% CIs from MR analyses (left), Cox proportional hazard models (middle, two different adjustment sets), and logistic regression models on prevalent disease status (right). Colors indicate the level of support as in (A). Relevant summary statistics can be found in Table S11.

We observed 193 protein-disease pairs with high-confidence genetic support, of which less than a quarter (*n* = 52) showed directionally concordant—and at least nominally statistically significant—support in biomarker studies (Figure 6A). A similar number of protein-disease examples (*n* = 59) showed statistically significant support but with opposing effect directions. The latter might be explained by compensatory mechanisms, but it exemplifies the need to contextualize purely genetically inferred “causality.” Conversely, among the 52,887 protein-disease associations passing statistical significance (*p* < 9.3 × 10^−8^) in the survival analysis, only 0.06% (*n* = 33) had directionally concordant, high-confidence support from genetic analysis, and another 36 had significant but directionally discordant evidence (Figure 6A; Table S11). The overlap between the two approaches did not improve, and even led to a loss in, convergent examples when accounting for potential confounders in survival analyses or considering prevalent cases.

For 44 protein-disease pairs, we identified coherent evidence from genetic, prospective survival, and prevalent disease analyses, suggesting that those protein biomarkers may not only be relevant for the onset of the disease but also its persistence (Figure 6B). Among those, plasma furin levels stood out, being consistently associated with hypertension, myocardial infarction, and arterial fibrillation (Figure 6). The genetic locus encoding furin was first linked to blood pressure^39^ and later to cardiovascular diseases, with recent proteomic studies also confirming our results in another ancestry.^40–42^ Furin is an endopeptidase expressed in almost all tissues, with expression highest in the liver and with a broad substrate profile relevant for cardiovascular disease. Our results collectively point to the role of extracellular rather than intracellular furin. Almost all previously reported furin effects were described to occur in the *trans*-Golgi network, and extracellular furin has been mostly investigated in relation to the cleavage of pathogen products.^43^ The convergence of genetic and survival analysis (Figure 6B) may provide evidence for an extracellular role of furin in disease etiology, in contrast to the hypothesis that associations with the risk of hypertension and coronary artery disease are mediated via intracellular cleavage of brain natriuretic peptide (BNP)^44^ or modulation of the LDL-receptor and cholesterol metabolism.^45^ This hypothesis is supported by a lack of association between the lead *cis*-pQTL for furin (rs8027450-T) and circulating levels of both BNP (beta = 0.006; *p* = 0.2) and N-terminal prohormone of BNP (NT-proBNP, beta = 0.0058; *p* = 0.3). Pharmacological inhibition of furin in mouse models provided preliminary evidence for reduced atherosclerotic lesions and reduced vascular remodeling.^46^

### *trans*-pQTL enrichment explains protein-disease signatures and can guide drug repurposing

The low convergence of evidence for protein-disease links from observational associations versus genetic inference from *cis*-pQTLs might, in part, be explained by circulating proteins being merely surrogates for causal disease processes within tissues. Investigating the value of many newly identified *trans*-pQTLs, we identified that >90% (280 out of 307 diseases with ≥5 significantly associated proteins) of disease biomarker signatures were significantly enriched (FDR < 0.05) for proteins associated with one or more of 170 pleiotropic pQTLs (*n* = 139 *trans*-pQTL; Table S12).

Triangulating the evidence from pQTLs with GWASs and biomarker analyses, we observed 58 examples where the protein targets associated with a given pleiotropic pQTL showed an enrichment for disease-associated plasma proteins, where the pQTL was also a GWAS locus for the same disease (Figure 6C). This supports the hypothesis that part of the circulating protein signature provides a readout of disease-predisposing processes within tissues.^47^ For example, we observed a >50-fold enrichment (OR = 56.3, FDR < 9.5 × 10^−20^) of proteins significantly associated with the onset of proteinuria among those proteins associated with a *trans*-pQTL, for which we prioritized *SHROOM3* as a candidate causal gene (Figure 6C; Table S7). Knockdown of the mouse equivalent *Shroom3* has been shown to induce proteinuria by affecting podocyte foot process effacement,^48^ impacting the cells forming part of the layer responsible for size-selective filtration of blood in the kidneys.

**Figure 7.**
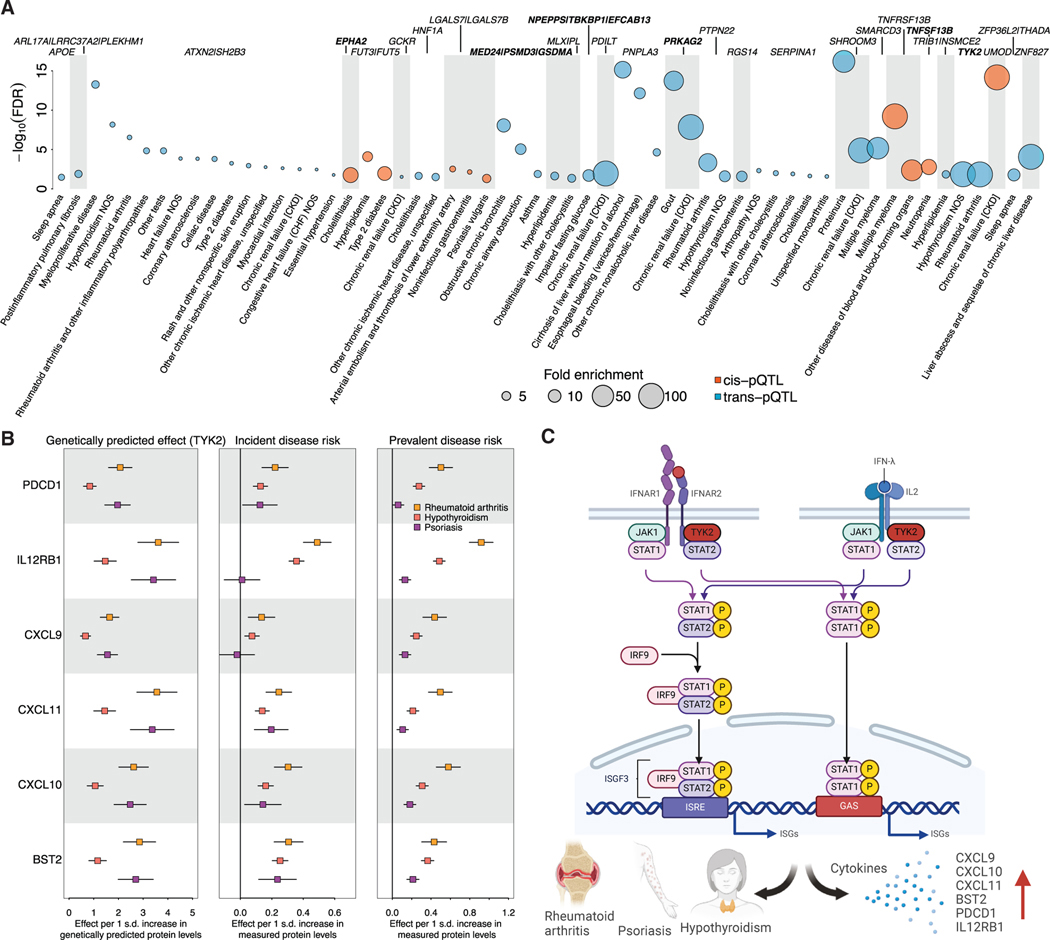
*trans*-pQTL enrichment explains prospective biomarker signatures and supports repurposing of TYK2 inhibitors (A) Protein biomarker signatures significantly (FDR < 5%) enriched for pQTLs that have been reported to associate with the risk of the respective disease. The most likely effector genes are annotated. Genes that are the target of drugs are highlighted in bold. (B) Forest plots showing associations (effect estimate with 95% CIs) between genetically predicted plasma protein levels (left; using rs34536443 in *TYK2*) and measured plasma protein levels (middle, right) with the risk/presence of psoriasis, hypothyroidism, and rheumatoid arthritis. (C) Schematic displaying a potential role of impaired cytokine signaling due to loss of function in TYK2 for cytokine secretion and the onset of different auto-immune diseases. This figure was created using BioRender.com. See also Figure S4.

Plasma protein signatures non-randomly linked to known disease variants may further guide drug repurposing and biomarker identification for patient selection. For example, a missense variant (rs34536443, p.P1104A) in tyrosine kinase 2 (*TYK2*) acted as a *trans*-pQTL for multiple proteins (bone marrow stromal antigen 2 [BST2], C-X-C motif chemokine 9 [CXCL9], CXCL10, CXCL11, interleukin-12 receptor subunit beta-1 [IL-12RB1], and programmed cell death protein 1 [PDCD1]) (Figure 7). Higher plasma levels of all six protein targets were associated with a higher disease risk for rheumatoid arthritis, hypothyroidism, and psoriasis. There was also strong evidence for a shared genetic signal across these proteins and disease risks (HyPrColoc posterior probability [PP] = 98.9%) at the *TYK2* locus (Figures 7 and S4). Psoriasis is the approved indication for the *TYK2* (encoded at *TYK2*) inhibitor deucravacitinib, which mitigates inflammatory burden in patients,^49^ supported by our genetic evidence (Figures 7 and S4). The associated circulating protein signature might therefore be indicative of early inflammatory dysregulation in patients at risk of these autoimmune diseases, and persistently elevated plasma levels among patients might help to identify those most likely to benefit from the treatment, as well as monitor treatment success, as demonstrated in a recent phase 2 trial of deucravacitinib.^50^ This example illustrates the potential value of *trans*-pQTLs to guide biomarker prioritization for diverse purposes, benefiting patients as well as informing drug repurposing opportunities.

## DISCUSSION

Broad-coverage, high-throughput proteomic technologies applied to large-scale patient and population cohorts offer a comprehensive molecular view that can substantially deepen our understanding of human health and disease.^6–24^ Here, we present large-scale antibody-based proteogenomic meta-analyses consisting of 38 cohorts and up to 78,664 individuals to derive an expansive pQTL catalog of 24,738 fine-mapped pQTLs (including 5,040 in *cis* and 19,698 in *trans*). The multi-cohort design of our study provides consistent evidence across cohorts for >80% of the pQTLs identified in this study and also demonstrates that cohort and sample characteristics contribute only relatively minimally to observed heterogeneity of effects, in comparison to the overall strength of association, i.e., genetic effect size.

We demonstrate the importance of distal (i.e., *trans*) genetic regulation for circulating protein targets by identifying pathways, tissues, and cell types with specific, but also broader, roles. By integrating effector gene assignments across protein targets, we were able to reconstruct entire pathways relevant to plasma protein abundances through a data-driven approach based on in-human evidence. N-linked glycosylation emerged as the most frequently enriched pathway, in line with its role in the secretory pathway, involving the correct folding, trafficking, and secretion of the protein into blood or other tissues.^51–53^ Enzymatic protein glycosylation is the most abundant posttranslational modification, and our observation of enriched members of N- but not O-linked (a hallmark of intracellular proteins) glycosylation reflects a general finding of our study: that *trans*effector genes are more likely to be directly linked to protein targets actively secreted into the blood.

Many studies now screen for novel biomarkers or drug targets using publicly available data, either with individual-level access to resources such as UKBB or with summary statistics from large genetic consortia, the latter being pursued purely *in silico* with strong underlying assumptions. The discrepancies in the inference arising from both approaches may explain some of the limited visible success to date. Even the high-confidence examples identified here, such as furin’s role in diverse cardiovascular diseases, require follow-up before being evaluated as potential drug targets. We note that clinically useful protein biomarkers do not need to have genetic evidence to imply a causal role of the protein in disease development, but a clear and well-understood link to disease-predisposing mechanisms is essential for successful clinical application (e.g., neurofilament light chain used to diagnose neurodegenerative diseases).

By integrating different lines of orthogonal evidence, we provide clear examples of *trans*-pQTLs guiding biomarker prioritization for specific diseases. As a proof of principle, our results highlight seven *trans*-pQTLs directly supporting the use of NT-proBNP as a biomarker for (future) heart failure, with all *trans*-variants being associated with both heart failure and plasma NT-proBNP levels. Similarly, we exemplified how *trans-*pQTLs, when integrated with prospective biomarker analysis and large-scale GWASs of different diseases, can point to protein biomarkers to support drug repurposing, such as TYK2 inhibitors for rheumatoid arthritis.^54^ Collectively, this provides clear conceptual advances beyond *cis*-focused causal inference techniques, which have predominantly been applied in proteo-genomic studies to date, highlighting how *trans*-pQTLs can inform protein biomarker detection for diseases and drug development as well as improve our understanding of identified susceptibility loci for these diseases.

MR has become commonly used for genetically guided drug-target discovery. We observed discordant effects for well-curated *trans*-pQTL versus biologically plausible *cis*-pQTL disease associations. Although we acknowledge that limitations might remain for specific examples, our results did not reveal a systematic reason explaining the convergence or segregation of *cis* and *trans* effects on the diseases studied, and we propose a staged approach to guide the evaluation of *trans*-pQTLs in the context of MR studies. This includes biologically informed exclusion of instruments with evidence for pleiotropic effects on either the proteome or phenome, in addition to testing the alignment of *cis*- and *trans*-pQTL effect sizes and directions.

Our study demonstrates the potential of larger multi-cohort studies in expanding our understanding of the genetic regulation of the circulating proteome and of the insights into disease mechanisms and therapeutic development that proteogenomics can deliver.

### Limitations of the study

All proteomic technologies currently only provide a partial coverage of the proteins detectable in circulation, including their many isoforms and post-translational modifications. Similarly, we had incomplete coverage of the range of genomic variants across cohorts, as most cohorts had array-based imputed genomic data, limiting our ability to assess rare variants and utilize statistical approaches such as fine-mapping to their full extent. Our study was conducted using predominantly European samples due to measurement availability. We believe our study provides not only a valuable multi-cohort proteogenomic resource to the community but also a template for the biological and clinical insights that can be derived from such efforts, including future studies with even greater power, allele and protein coverage, and genomic diversity.

### RESOURCE AVAILABILITY

#### Lead contact

Further information and requests for resources should be directed to Claudia Langenberg (claudia.langenberg@qmul.ac.uk).

#### Materials availability

This study did not generate any new, unique reagents.

#### Data and code availability

- Access to the UKBB genomic, proteomic, and phenotype data is open to all approved health researchers (http://www.ukbiobank.ac.uk/). This research has been conducted using the UKBB resource under application no. 44448.
- Genome-wide summary statistics for all protein targets in this study will be available on https://omicscience.org/ upon publication.
- All remaining data have been accessed via publicly available links provided in the key resources table.
- Associated code and scripts for the analyses are available at https://github.com/comp-med/scallop-ukbb-ma.

## STAR★METHODS

### EXPERIMENTAL MODEL AND STUDY PARTICIPANT DETAILS

We performed a multi-cohort meta-analysis of genome-wide summary statistics of plasma levels of 1,194 protein targets from up to 37 cohorts (Table S1), referred to as SCALLOP meta-analyses. The participants of these cohorts were predominantly of European ancestry. Detailed information about the participant characteristics in each cohort, including mean age, mean BMI, percentage of females in the cohort and fasting status can be found on Table S1. All participants gave their informed written consent before entering the studies and each study was approved by their respective ethics committee.

We additionally included proteogenomic analysis conducted using genomic and proteomic data from 48,017 participants of European ancestry in UK Biobank (UKBB). Further information about the UKBB cohort and proteomic measurements can be found elsewhere.^20,55^

### METHOD DETAILS

#### Proteomic measurements

For the 37 cohorts included in this study, antibody-based proteomic measurements were generated through at least one of the 13 Target-96 panels offered by Olink measuring 92 protein targets (Cardiometabolic, Cardiovascular II, Cardiovascular III, Cell Regulation, Development, Immuno-oncology, Inflammation, Immune Response, Metabolism, Neurology, Neuro Exploratory, Organ Damage, Oncology II). Details regarding the assay have been described in detail elsewhere.^61,62^ Briefly, dual antibody based proteomic measurements are generated where each of the unique antibodies are labelled with complementary single stranded oligonucleotides, referred to as proximity extension assays (PEA).^61^ Hybridization occurs between the complementary oligonucleotides when both of the unique antibodies bind to their respective protein target and come into close proximity, which can subsequently be quantified through qPCR or next-generation sequencing (NGS) methods. Relative proteomic measurements are offered as normalized protein expression (NPX) units, provided on log2 scale. Each cohort has performed quality control on their proteomic measurements such as, but not limited to, removing samples that were extreme outliers using principal-component analysis from their entire proteomic profiles. Only blood-based (i.e. plasma or serum) proteomic measurements were included in this study.

In UKBB, the proteomic measurements used in this study were generated through Olink Explore 1536 platform utilizing the same antibody-based technology but measuring a broader coverage of 1,463 protein targets. Details of proteomic measurements can be found elsewhere.^20^

#### Genome-wide meta-analyses of protein levels

For 1,194 unique protein assays for each protein target from all 37 cohorts, we performed additional quality control measures and retained only biallelic variants with (i) a call rate above 95%, (ii) Hardy-Weinberg p-value above 1×10^−5^ , (iii) imputation INFO score above 0.8, (iv) standard error of SNP-effect less than 10, (v) minor allele count (MAC) above 3 and (vi) minor allele frequency above 0.1%. We filtered out any variant which did not have an existing rsID in the dbSNP database.^63^ Using all available summary statistics per protein target, we performed inverse-variance fixed-effects meta-analyses using METAL (v.2011–03-25),^59^ also referred to as SCALLOP meta-analyses. The sample sizes varied from 2,297 to 31,190 depending on protein target coverage across cohorts.

In parallel, we have also conducted genome-wide association analyses for proteomic measurements of 1,463 protein targets in UK Biobank participants through REGENIE v.3.1.4.^58^

Finally, we performed an inverse-variance fixed-effects meta-analyses (with METAL^59^) for 1,161 protein targets (with overlapping UniProt ID across different antibody-based platforms offered by Olink) using summary level data from SCALLOP meta-analyses and UKB, reaching to a total sample size of up to 78,664 participants (*n*=17,602 – 78,864).

#### Downstream analyses

Following regional clumping for independent signal selection, we applied Bayesian fine-mapping to identify independent protein quantitative trait loci (pQTLs) using SuSie.^27^ We characterized variance explained by significant pQTLs and heritability by polygenic background (excluding any significant loci for a given protein) through LD-score regression implemented in LDSC.^64^ Leveraging the multi-cohort study design, we assessed the confidence for of each pQTL by their (i) statistical significance, (ii) directional concordance across cohorts and (iii) observed heterogeneity. We then tested the contribution of participant and cohort-level characteristics to the heterogeneity observed through a meta-regression model. We used zero-inflated Poisson regression models to test for associations between the presence and number of associated cis-/trans-pQTLs per protein and various protein characteristics.

Using the output from machine learning models we trained for trans-pQTL effector gene assignment, we tested enrichment of the assigned effector genes among pathways, tissues, cell-types. Based on assessing the overlap of pQTLs or proxies (r^2^ >0.8) with phenome-wide associations from GWAS catalogue,^30^ we defined three categories of pleiotropy, (i) ‘molecular pleiotropy’ (associated with >5 proteins and ≤ 5 non-proteomic phenotypes), (ii) ‘phenotypic pleiotropy’ (associated with >5 non-proteomic phenotypes and ≤ 5 proteins), and (iii) ‘unspescific pleitropy’ (associated with >5 proteins and >5 non-proteomic phenotypes).

For phenotypic follow-up, we first assessed the link between cis-pQTLs with 835 diseases from the FinnGen^35^ release 8 using fine-mapping augmented colocalization^65^ and two sample MR analyses.^66^ We further systematically tested for the relevance of transpQTLs by adopting the cis-MR workflow and testing for (i) cis and trans-pQTLs and (ii) trans-pQTLs only as genetic instruments for each protein target. To test for concordance between genetically inferred protein – disease relationships and plasma protein levels and disease onset and/or presence, we first run Cox-proportional hazard models and logistics regression models, respectively, adjusting for age, sex, and technical variables. We then integrated cis-based two-sample MR analyses from an internal pan-biobank project covering over one million individuals (MVP,^67^ the Pan-UK Biobank,^68^ and FinnGen^35^) and tested for concordance between the genetic and observational approaches using the relevant multiple-testing corrected significance thresholds.

Finally, we tested whether the protein association profile of pQTLs that were reported for at least one non-proteomic trait in the GWAS Catalog were significantly enriched for any pathway and whether there was a non-random overlap between protein-biomarker signatures from prospective biomarker analyses and protein signatures associated with pQTLs using Fisher’s exact test. Detailed methods can be found on quantification and statistical analysis section.

### QUANTIFICATION AND STATISTICAL ANALYSIS

#### Genome-wide meta-analyses of protein levels

We did not observe evidence for genomic inflation for (i) SCALLOP only meta-analysis (mean=1.04, IQR=1.04–1.05) or UKBB proteogenomic analyses (mean=1.09, IQR=1.07–1.10).

Using the summary statistics from SCALLOP meta-analyses and UKBB proteogenomic analysis, we performed inverse-variance fixed-effects meta-analyses using METAL (v.2011–03-25)^59^ for 1,161 protein targets, mapping to the same UniProt ID. We also did not see evidence for genomic inflation across the overall meta-analyses (mean=1.08, IQR=1.06–1.09). Genomic build GRCh37 was used throughout this study.

#### Identification of regional sentinel variants

Regional sentinel variant selection was performed by selecting the variant with the highest z-score within each 1 Mb window around each significant signal. The genomic regions were merged and considered as a single region if there were multiple significant variants within less than 500 kb from each other. Bonferroni corrected genome wide significance thresholds were used for each sub-study as follows, p<4.19×10^−11^ for SCALLOP meta-analyses (1,194 protein targets), p<3.42×10^−11^ for UKBB proteogenomic analyses (*n*=1,463 protein targets) and p<4.31×10^−11^ for the overall meta-analyses (*n*=1,161 overlapping protein targets).

pQTLs were defined as cis-acting if they were located within 500 kb of a protein’s cognate gene and were defined as trans-acting otherwise. Where assays targeted more than one protein, *cis*-pQTLs were defined as those located within 500 kb of any potential cognate genes.

#### Fine mapping

We performed statistical fine-mapping on protein GWASs performed in the UKBB, as the largest single study, using the ‘sum of single effects’ model (SuSiE).^27^ Briefly, SuSiE implements variable selection under a Bayesian framework to identify credible sets of independent variants that likely contain the true underlying causal variant. We conducted these analyses using the R package susieR (v.0.12.16) with the default parameters and priors. We used a random subset of 20,000 unrelated participants of European ancestry from the UKBB as an LD reference. Additionally, we adopted a grid search approach that iterated the maximum number of credible sets from 2 to 10, selecting the number that ensured no variants within a credible set were in LD (r2> 0.1). Credible sets were taken forward where variants additionally met genome-wide significance. We have included regional summary statistics from the overall meta-analysis for any region where it was not possible to perform SuSie on UKBB summary statistics or regions on X-chromosome. We also clumped the independent credible sets into r2 groups across all protein targets based on linkage disequilibrium (r2>0.8).

#### Concordance of the identified pQTLs

Variants were characterized as ‘high confidence’ if the pQTL was (i) at least genome-wide significant (p<5×10^−8^), (ii) showed directional consistency in the overall meta-analyses between SCALLOP cohorts and UKBB for *cis*- and *trans*-pQTLs and (iii) additionally did not show substantial evidence of heterogeneity (p_het_ >1×10^−4^) for *trans*-pQTLs.

We tested for independent replication of identified lead variants from credible sets by investigating for the overlap for the pQTLs or any of their proxies (r2> 0.1) in previously published affinity-based proteogenomic studies, based on a common UniProt ID for the protein targets.^6–24^

We tested the correlation between the effect size for regional sentinel variants identified in this study with cohorts of predominantly European ancestry with effect size of the pQTL in cohorts of non-European ancestry, namely Immuno-oncology panel in China Kadoorie Biobank (CKB, *n*=816 participants of Chinese ancestry), Metabolism panel in Qatar Metabolomics study on Diabetes (QMDIAB, *n*=350 participants of Indian, Filipino or Arabic ancestry) and Inflammation, Cardiovascular III, Neurology and Oncology II panels in Shanghai Women and Men’s Health Study (SWMHS, *n*=548 participants of Chinese ancestry) (Figure S2).

#### Variance explained by identified pQTLs

We estimated the variance explained by the regional sentinel variants and heritability estimates by the polygenic background, excluding the regions harbouring any pQTLs^18^ for all 1,161 protein targets included in this study. In summary, we calculated variance explained for cis and trans loci by using the formula (2 × β2 × f × (1−f)), where f is the MAF. We calculated heritability estimates by the polygenic background, by excluding the regions harbouring a pQTL, through LD-score regression (LDSC).^64^

#### pQTLs and protein-related characteristics

We tested the association of presence and also the number of high confidence cis- or trans- pQTLs with a wide range of protein-related characteristics, namely whether a protein (a) has sites for sulfation, glycosylation, phosphorylation, ubiquitination, s-nitrosylation, acetylation, palmitoylation, acetylglucosmination, glycosaminoglycan-chains, myristoylation, acylation and methylation, (b) has any disulfide bonds, DNA binding domains, alpha-helix domains, turn domains, transmembrane domains, zinc finger domains, coiled coil domains, beta-strand domains, or a protein’s (c) transcript count as obtained from UniProt.^57^ We also tested the association of the presence and also the number of high-confidence cis- or trans-pQTLs with the probability of loss of function intolerance (pLI), missense Z-score (i.e. the deviation of the observed from the expected number of missense variants), protein length and gene length as obtained from gnomAD v2.1.^56^ We used ‘pscl’ package in R v.4.2.2 which performs zero-inflated Poisson regression characteristics by including scaled sample sizes as a covariate. In summary, the package runs two set of tests for each characteristic which are (i) Poisson regression for the count data to test the association between the number of high-confidence cis or trans pQTLs and protein-related characteristics, (ii) logistic regression to test the association between the existence of high confidence cis or trans pQTLs and protein-related characteristics. The continuous traits were normalized to have a comparable effect size estimate with the binary ones. We applied a Bonferroni-corrected significance threshold of p<2×10^−3^ (corrected for the number of investigated characteristics, *n*=25). We also ran sensitivity analyses including missingness rate for proteins as a covariate in the model.

#### Impact of cohort characteristics on heterogeneity

To better understand cohort characteristics that might contribute to the heterogeneity, we performed meta-regression analyses in R v4.2.2 using ‘metafor’ package. The loci where we observed high levels of heterogeneity (heterogeneity p-value <1×10^−4^) across SCALLOP cohorts from the fixed-effects meta-analyses were taken forward and a meta-regression model was fit with between the effect size of these loci from each cohort and cohort characteristics and the overall genetic effect size of the loci (from the meta-analysis) as explanatory variables. The cohort characteristics that were included were mean cohort age, sex composition of the cohort (i.e. %females), mean BMI of the cohort, blood-based tissue type (EDTA plasma, citrate plasma, or serum), fasting status and whether any disease cases were included in the cohort (Table S1).

#### Characterization of variant consequences

We used Variant Effect Predictor (VEP, version 99) through Ensembl to characterize the consequence of the fine-mapped variants and their proxies.^60^ We used Graphql to query the Opentargets platform^69^ to obtain the closest gene for each unique fine-mapped pQTL variant.

#### Colocalization with gene expression levels

We systematically tested for a shared genetic signal between plasma abundance of a protein and gene expression levels (eQTL) of the protein coding gene in 49 tissues from the GTEx project (v8)^25^ assessed by posterior probability from statistical colocalization analysis using ‘coloc’ package in R 3.6.^70^ The build of our study was lifted over from b37 to b38 using LiftOver,^71^ to be compatible for the analyses. If any of the pQTLs did not have a corresponding genomic location in b38, they were excluded from the downstream colocalization analysis. GTEx^25^ variant-gene cis-eQTL associations from each tissue were downloaded from https://console.cloud.google.com/storage/browser/gtex-resources on January 2020.

For the cis-variants, the region was defined as (±500 kb) around the protein-encoding gene of each protein-encoding gene with at least one *cis*-pQTL that were present in both datasets. For *trans*-pQTL - *cis*-eQTL colocalization analyses, the region was defined as the ±500 kb window around the *trans*-pQTL signal, regardless of the genes that reside in the region. The colocalization analysis was only performed if the region had at least suggestive evidence of association (p<10^−6^) in the defined region in any of the tissues and the lead variant was in high LD with the lead *cis*-pQTL in the region (r^2^ >0.6). A prior probability of a shared signal (p12) was defined as 1×10^−5^ and posterior probability above 80% was used to define a high likelihood of a shared genetic signal.

#### Machine learning-based effector gene assignment

We implemented a staged machine learning classifier to systematically assign candidate effector genes for all *trans*-pQTLs residing outside of the MHC region. We first collated comprehensive annotations for each genetic variant or proxies thereof (r^2^ >0.6), including 1) distance to the gene body in 1Mb window, and 2) putative functional consequences based on the variant effector prediction (VEP) tool. We further systematically collated for each gene within a 1Mb window: 1) evidence for colocalization between gene and protein expression levels based GTEx version 8,^25^ 2) evidence for a rare gene burden association based on Dhindsa et al. (2023),^22^ 3) evidence whether any trans gene encodes for receptor/ligand or protein complex of the cis-protein based on literature evidence using the OmnipathR package v3.10.1,^72^ and 4) whether any of the genes participate in the same biological pathway based on KEGG^73^ and REACTOME^74^ annotations.

In the absence of generalisable gold-standard variant to gene assignments, we leveraged prior biological and genomic knowledge to derive three partly distinct sets of ‘putative true positive’ (PTP) sets: 1) Trans genes that encode ligand – receptor pairs of those with high-confidence evidence for forming a protein complex with the cis-protein (*n*=540 PTPs), 2) sentinel trans-pQTLs mapping to functional variants (*n*=1,747 PTPs), and 3) significant gene burden results for trans genes (*n*=1,049 PTPs). For each of the three PTP sets, we considered all other genes within a 1Mb window in the locus as negative examples. We pruned each set to contain at each locus only one cis-protein to avoid artificially good results due to pleiotropy. We then split each of the data sets 10 times in a 7:3 ratio to obtain training and test sets separating by genomic region. For each of the ten training sets, we trained a Random Forest classifier using repeated 3-fold cross-validation implementing subsampling to account for the unbalanced data sets. This was implemented using the R caret v6.0.94 package. We used the Kappa score to select the best performing forest within each training set. Eventually, we used each of the ten Random Forest classifiers from each PTP set to assign candidate scores for all putative effector genes across the entire set of trans-pQTLs. We thereby took the median score across all ten classifiers for a PTP set and finally summed the score across all three predictions. For each of the PTP sets, we omitted features used to define true positive sets. All three classifiers showed robust performance with median Kappa values of 0.54 – 0.57. The conception of our machine learning classifier was inspired by the ProGeM framework^75^ and with a rationale of integrating biologically relevant annotations such as ligand – receptor pairs or protein complexes.

#### Pathway, tissue, and cell-type enrichment

We obtained processed data for tissue and single cell expression from the Human Protein Atlas,^76^ and used the annotations ‘specific’ and ‘enhanced’ to define a common set of genes for each tissue/cell-type with enhanced expression. We used Fisher’s exact tests to test for a significant enrichment of assigned effector genes among enhanced gene expression sets. To avoid redundancies, we only took the effector gene with the highest score at each locus to test for enrichments. If required, we restricted the background of those tests to proteins covered on the Olink panels and computed the false discovery rate to account for multiple testing. We report only enriched, but not depleted tissues/cell-types, although those have been considered when performing multiple testing correction. For pathway enrichment, we used the R package gprofiler2 v0.2.2 restricting KEGG^73^ and REACTOME^74^ as data bases and FDR-correction.

#### Phenotypic follow up

We used two partially complementary approaches to systematically link *cis*-pQTLs to 835 diseases from the FinnGen release 8.^35^ Firstly, we used fine-mapped protein GWAS summary statistics to colocalize credible sets in ±500kB windows around protein encoding genes, as implemented in the R package coloc with a recent augmentation to relax the single variant assumption using the SuSiE method.^65^ Colocalization was only performed if there was at least a suggestive signal for a given region for the trait being tested. We used default priors apart from implying a more stringent prior to declare a shared genetic signal (p_12_ =5×10^−6^). To fine-map FinnGen outcomes, we used at most 5 credible sets and a random set of 30,000 UKBB participants to estimate an LD-backbone. We considered evidence of a shared signal between a protein and a FinnGen outcome, if at least one credible set was shared among both traits (PP H4 > 80%) provided the lead *cis*-pQTL was also in LD with the fine-mapped lead signal for the FinnGen trait. We used simple Wald ratios to assess effect directions, i.e., to understand whether an increase in protein levels is associated with an increase/decrease in disease risk. Secondly, we performed standard cis-based MR analysis,^66^ by aggregating Wald ratios across all independently selected, genome wide significant *cis*-pQTLs and any of the FinnGen outcomes using the inverse variance weighted method. We required overlap of at least two-thirds of identified *cis*-pQTLs to avoid artificial findings. Apart from correcting for multiple testing using Bonferroni correction, we further required consistent effect directions in sensitivity analysis, including weighted median, mode, and MR-Egger regression, as well as no strong evidence for heterogeneity (p>10^−3^) or pleiotropy (p>10^−3^).

We further systematically tested for the relevance of *trans*-pQTLs by adapting the *cis*-MR workflow as follows. We considered all high-confidence *trans*-pQTLs associated with a protein target that were associated with less than five protein targets or phenotypes in the GWAS Catalog,^30^ i.e. specific *trans*-pQTLs. We first identified instruments strongly violating MR assumptions by performing leave-one-out analysis tracking Cochran’s Q statistic and omitted any instrument accounting for strong (median + 3 X SD) deviations compared to all other combinations of instruments - a procedure similar to recently proposed, but computationally more expensive, methods.^77^ We also tested combining cis- and *trans*-pQTLs. Similar to the *cis*-MR, we applied the Bonferroni correction to account for multiple testing and implemented several sensitivity analyses including performing MR-Egger to assess pleiotropy, assessing directionally concordance the across different MR methods and assessing the heterogeneity among the pQTLs. We further demonstrated the need to account for pleiotropy via biological insights by running MR analyses without pleiotropy filters for trans-pQTLs.

#### Cis-based pan-biobank phenotypic follow up

To harmonize phenotypes between MVP,^67^ the Pan-UK Biobank,^68^ and FinnGen^35^ (version 10), disease-based traits were mapped using codes provided by the biobanks. MVP used entirely phecodes (*n*=1,171), while the UKBB used phecodes (*n*=1,327) as well as ICD10 codes (*n*=915) to label their disease phenotypes. After restricting studies in the UKBB to those with European ancestry in their list of populations, all possible direct phecode to phecode (n=1,013) matches were made between the biobanks. UKBB traits with an ICD10 code were then mapped to phecodes using a conversion table (70), and if the derived phecode had not already been mapped to MVP in the previous step, then a match was made if possible (n = 10). For MVP phecodes that remained unmapped to UKBB, studies with case counts higher than 4,000 were reviewed and a decision was made on a case-by-case basis to map manually (n = 53) by a clinician before aligning with FinnGen. For all unmapped MVP phecodes with case counts lower than 4,000, MVP only data was used for analysis. To map MVP and UKBB phenotypes to FinnGen, we manually mapped all available R10 FinnGen phenotypes to those previously available from MVP or UKBB. All matches were double checked through clinical adjudication and comparison statistics using the number of cases per resource for each phenotype to identify significant outliers from this mapping strategy. Situations where a significant deviation from the overall relationship between MVP/UKBB/FinnGen case counts was observed were analyzed on a one-by-one case and a final harmonization decision was made. This led to FinnGen being mapped only with MVP (32 instances), only with UKBB (174 instances), and with both MVP and UKBB (501 instances).

Pan-UK Biobank GWAS physical positions were converted from genome build GRCh37 to GRCh38 using LiftOver.^71^ Using METAL,^59^ we performed fixed effects inverse-variance weighted meta-analyses of MVP European results, UKBB European results, and FinnGen and obtained estimates of heterogeneity (option in METAL: ANALYZE HETEROGENEITY). If mapping was not possible between MVP, UKBB, or FinnGen, then only MVP results or select UKBB phenotypes were retained for further analyses. For very small p-values in MVP which were set to zero, we set to the lowest possible decimal place allowed in Python using sys.float_info.min() (2.2250738585072014e-308). To test for inflation of p-values following meta-analyses, we calculated the lambda values for all meta-analyses results. Any phenotype having a lambda value greater than 1.15 (n = 289) was rerun including the genomic control parameter in METAL. Phenotypes that were not meta-analyzed were also tested for inflated p-values. We found 81 phenotypes that needed correction, the vast majority being clinical biomarkers (75 studies). Due to highly inflated p-values, we removed eight height-based phenotypes from consideration.

Two-sample Mendelian Randomization (MR) of each of the protein-coding genes were performed against all phenotypes using instruments from SCALLOP *cis*-pQTLs. In order to determine the correct ordering of alleles between the datasets we utilized the harmonise_data() function from the TwoSampleMR^78^ package in R.

We used the Wald Ratio for instruments with one genetic variant and inverse variance weighted MR for instruments with multiple genetic variants. We additionally performed MR-Egger for proteins with three or more instruments to be used as a sensitivity analysis. We tested for heterogeneity across variant-level MR estimates, using the Cochrane Q method (mr_heterogeneity option in TwoSampleMR package) and the MR-Egger intercept.

#### Comparison of observational and genetic analyses

To test for concordance between genetically inferred protein – disease relationships and plasma protein levels and disease onset, we performed two analysis streams. We collated electronic health records, such as primary (45% of the population) and secondary care, death certificates, or cancer registry, but also self-reported information into 1,448 medical ontology terms called ‘phecodes’ (which we refer to as ‘diseases’ for simplicity) for all UKBB participants.^79^ We kept track of the first occurrence of each disease code in any of the resources and classified participants that had any code for a given disease prior to inclusion into UKBB or reported it at recruitment as ‘prevalent’, and any disease occurrence thereafter as ‘incident’. We then associated plasma protein levels with the risk of disease onset using Cox-proportional hazard models with age as the underlying timescale while adjusting for sex of the participant and technical variables, such as sample age and fasting time. We monitored the proportional hazard assumption using Schoenefeld residuals and applied different levels of multiple testing correction to declare significance (see below). For each disease outcome, we excluded participants with codes indicating prevalent diseases. We only tested diseases with at least 10 cases (*n*=765) among all 52,169 participants with overlapping proteomic and disease data. Secondly, we integrated the genetic analyses from pan-biobank genetic analyses as described above and restricted those protein–disease associations available in both pan-biobank genetic analyses and prospective analyses. For genetic discovery, we defined high confidence protein-disease associations as those passing correction for multiple testing across protein-disease pairs and with support from colocalization (PP4 > 0.8). We then sought support from prospective analyses for each high confidence protein-disease pair at p < 0.05. For prospective discovery, after correction for multiple testing, we investigated whether there was genetic support for each protein-disease pair at the maximum p-value where colocalization was performed (5×10^−6^) and PP4 > 0.8.

#### Enrichment analysis anchored on pQTLs

We performed two different sets of enrichment analysis utilizing pQTLs. Firstly, for pQTLs reported for at least one non-proteomic trait in the GWAS Catalog,^30^ we tested whether significantly associated protein targets were enriched for pathways using the same settings as described above using the Gprofiler software but setting the background for enrichment testing to the proteins captured in our study. Secondly, we tested for a non-random overlap between protein biomarker signatures from prospective biomarker analyses and protein signatures associated with pQTLs using Fisher’s exact test. We used the proteins included in all analyses as a background and proteins that associated with disease onset and the trans-pQTL as foreground. We tested only for enrichments if at least five proteins were associated with the trans-pQTL. We linked enrichment analysis to prospective biomarker analysis further by mapping GWAS Catalog entries to phecodes to assign convergence.

## Supplementary Material

MMC11

MMC10

MMC8

MMC5

MMC2

MMC9

MMC4

MMC3

MMC6

MMC7

MMC1

MMC12

13

SUPPLEMENTAL INFORMATION

Supplemental information can be found online at https://doi.org/10.1016/j.cell.2026.03.049.

## Figures and Tables

**Table:** KEY RESOURCES TABLE

REAGENT or RESOURCE	SOURCE	IDENTIFIER

**Deposited data**		

Genome-wide summary statistics for 1,161 circulating proteins	This study	https://omicscience.org/
UK Biobank data	UK Biobank^55^	www.ukbiobank.ac.uk
FinnGen summary statistics	FinnGen Consortium^35^	https://www.finngen.fi/en/access_results
GTEx summary	GTEx Consortium^25^	https://gtexportal.org/home/downloads/adult-gtex/overview
gnomAD data	gnomAD Consortium^56^	https://gnomad.broadinstitute.org/data
UniProt	The UniProt Consortium^57^	http://uniprot.org/
GWAS Catalog	Sollis et al.^30^	https://www.ebi.ac.uk/gwas/

**Software and algorithms**		

R	The R Foundation	https://www.r-project.org/
REGENIE	Mbatchou et al.^58^	https://rgcgithub.github.io/regenie/
METAL	Willer et al.^59^	https://github.com/statgen/METAL
Variant Effect Predictor (VEP)	McLaren et al.^60^	https://www.ensembl.org/info/docs/tools/vep/

## Highlights

- Proteogenomic evidence from the largest multi-cohort (*n* = 38) study to date
- Identification of genes, pathways, and cell types that modulate circulating protein levels
- Circulating protein signatures with genetic evidence that inform drug repurposing
- Proteogenomic insights into mechanisms underlying diverse diseases
