# Supplementary material for: Multi-cohort proteogenomic analyses reveal genetic effects across the proteome and diseasome
Source: Cell. Author manuscript; Available in PMC 2026 Jul 8. (PMC13343581; doi:10.1016/j.cell.2026.03.049)
Supplement: 13 [file NIHMS2173841-supplement-13.pdf]

CellPress
OPEN ACCESS

Supplemental figures

A

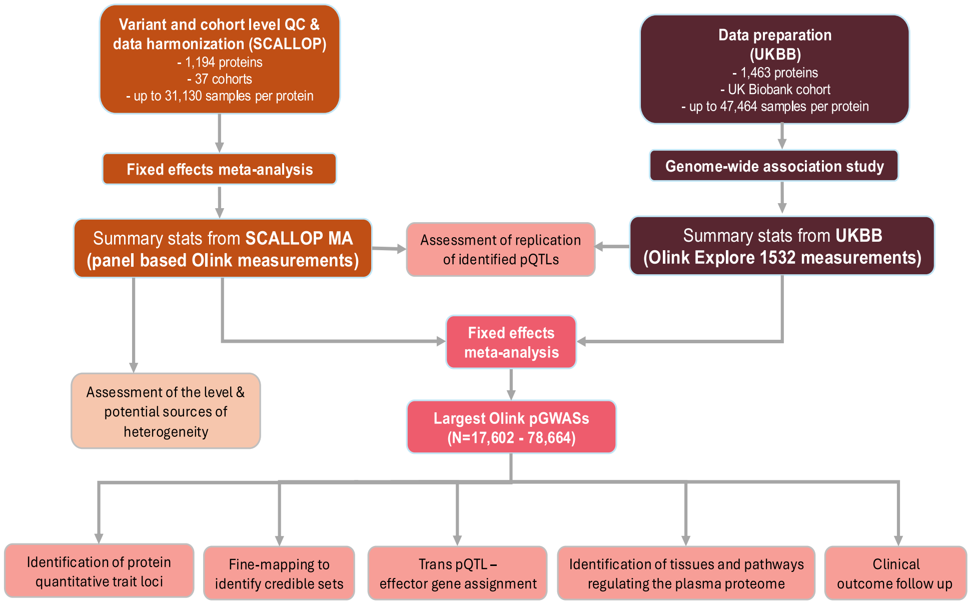

B

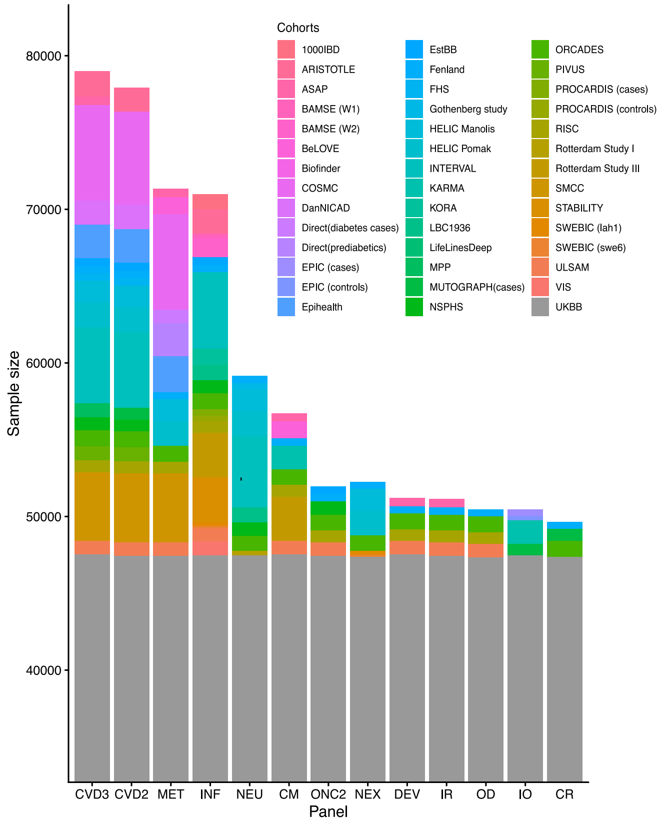

(legend on next page)

Figure S1. Overview of the study, related to Figure 1
(A) Study design.
(B) Distribution of sample sizes across cohorts and panels. The axis representing UKBB samples has been split for better visibility, and the *y* axis scale starts from 35,000 to 80,000.

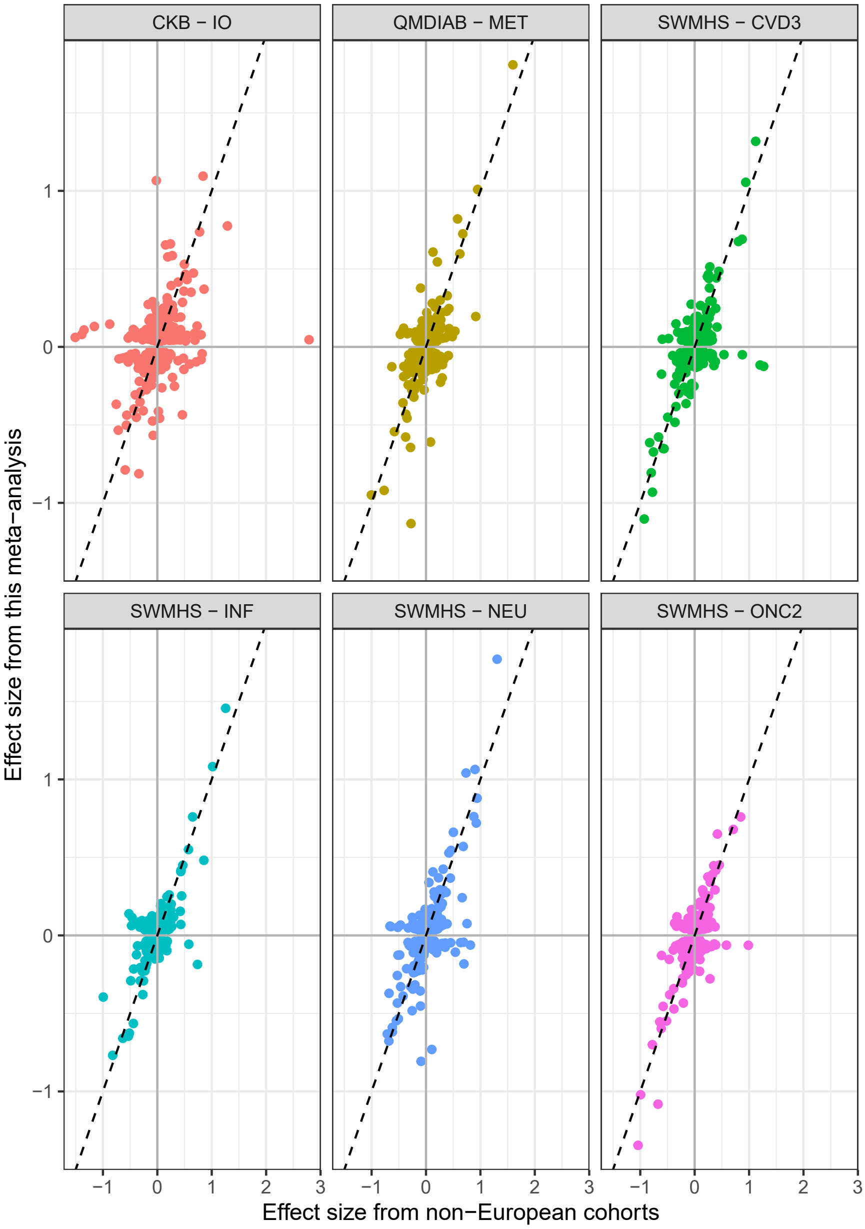

Figure S2. Correlation between the effect sizes of regional sentinel variants identified in this meta-analysis of participants mostly from European ancestry and cohorts of non-European ancestry for overlapping variant-protein pairs, related to Figure 1

The cohort-panel pairs visualized are the immuno-oncology panel in the China Kadoorie Biobank (CKB, n = 816 participants of Chinese ancestry), metabolism panel in the Qatar Metabolomics Study on Diabetes (QMDIAB, n = 350 participants of Indian, Filipino, or Arabic ancestry), and inflammation, cardiovascular III, neurology, and oncology II panels in the Shanghai Women and Men's Health Study (SWMHS, n = 548 participants of Chinese ancestry).

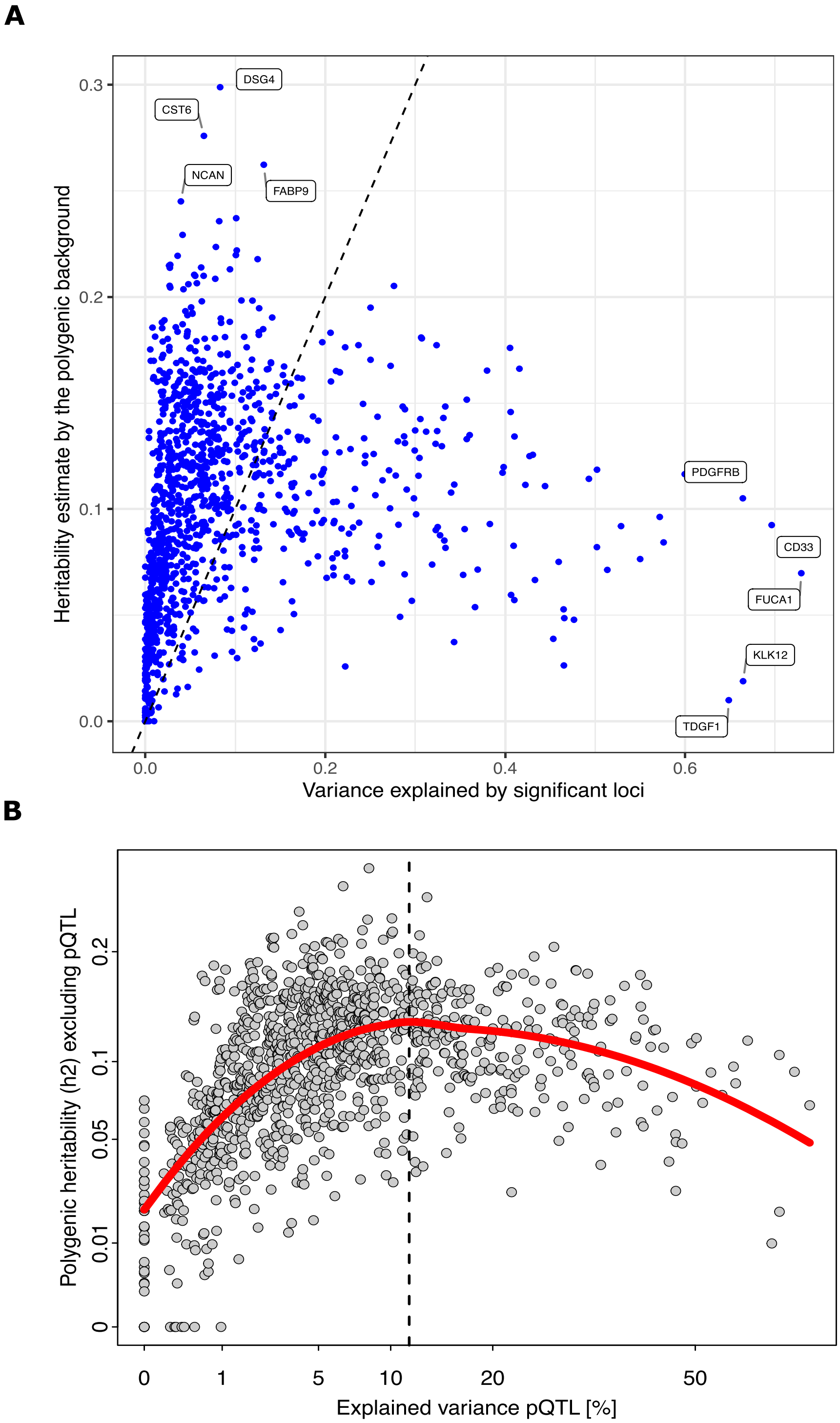

Figure S3. Variance and heritability estimates of 1,161 protein targets included in this study, related to Figure 1

(A) Variance explained by significant loci and heritability estimates excluding regions harboring any pQTLs for all 1,161 proteins included in this study.

(B) Scatterplot of protein targets with their square-root-transformed variance explained by significant loci plotted on the *x* axis and square-root-transformed heritability estimates, excluding regions harboring any pQTLs, plotted with a LOESS (locally estimated scatterplot smoothing) curve in red.

CellPress
OPEN ACCESS

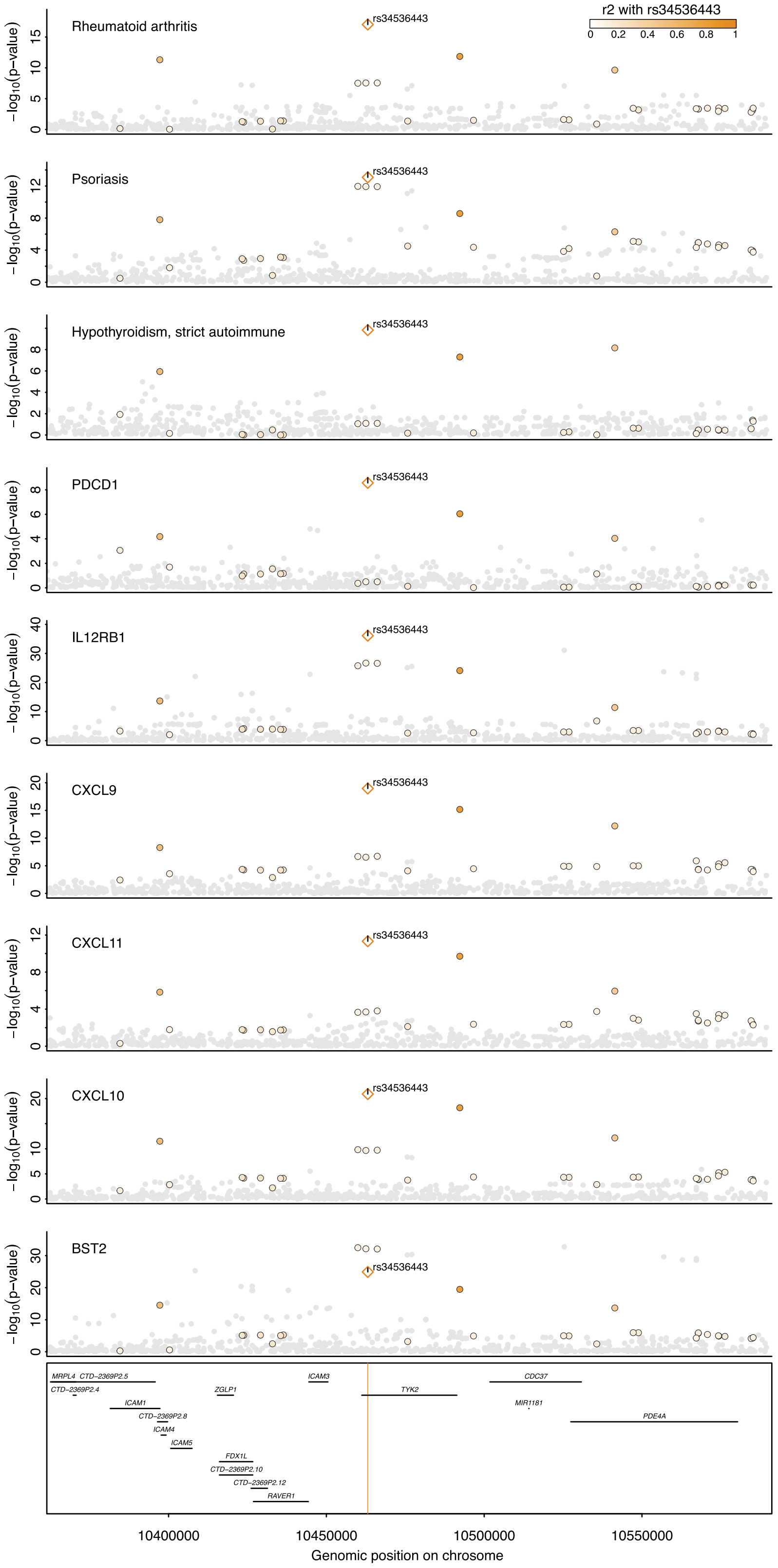

Figure S4. LocusZoom plot for *trans*-pQTL (rs34536443) and its association with multiple proteins (BST2, CXCL9, CXCL10, CXCL11, IL-12RB1, and PDCD1) and disease risk for rheumatoid arthritis, hypothyroidism, and psoriasis (HyPerColoc PP = 98.9%), related to Figure 7
